# One year of modeling and forecasting COVID-19 transmission to support policymakers in Connecticut

**DOI:** 10.1038/s41598-021-99590-5

**Published:** 2021-10-12

**Authors:** Olga Morozova, Zehang Richard Li, Forrest W. Crawford

**Affiliations:** 1 Program in Public Health, Department of Family, Population and Preventive Medicine, Stony Brook University (SUNY), Stony Brook, NY 11794 USA; 2 Department of Statisitcs, University of California, Santa Cruz, Santa Cruz, CA 95064 USA; 3 Department of Biostatistics, Yale School of Public Health, New Haven, CT 06510 USA; 4 Department of Statistics and Data Science, Yale University, New Haven, CT 06510 USA; 5 Department of Ecology and Evolutionary Biology, Yale University, New Haven, CT 06510 USA; 6 Yale School of Management, New Haven, CT USA

**Keywords:** Infectious diseases, Epidemiology

## Abstract

To support public health policymakers in Connecticut, we developed a flexible county-structured compartmental SEIR-type model of SARS-CoV-2 transmission and COVID-19 disease progression. Our goals were to provide projections of infections, hospitalizations, and deaths, and estimates of important features of disease transmission and clinical progression. In this paper, we outline the model design, implementation and calibration, and describe how projections and estimates were used to meet the changing requirements of policymakers and officials in Connecticut from March 2020 to February 2021. The approach takes advantage of our unique access to Connecticut public health surveillance and hospital data and our direct connection to state officials and policymakers. We calibrated this model to data on deaths and hospitalizations and developed a novel measure of close interpersonal contact frequency to capture changes in transmission risk over time and used multiple local data sources to infer dynamics of time-varying model inputs. Estimated epidemiologic features of the COVID-19 epidemic in Connecticut include the effective reproduction number, cumulative incidence of infection, infection hospitalization and fatality ratios, and the case detection ratio. We conclude with a discussion of the limitations inherent in predicting uncertain epidemic trajectories and lessons learned from one year of providing COVID-19 projections in Connecticut.

## Introduction

Epidemiologic models of infectious disease transmission have played an important role in supporting public health decision-making during the COVID-19 pandemic^1–7^. By specifying structural features of infection transmission dynamics, models can provide insights into epidemiologic parameters, historical trends in epidemic dynamics, or future outcomes under hypothetical intervention scenarios. Transmission models are especially useful in situations of high uncertainty, offering a structured way to assess the potential effects of interventions given plausible assumptions about disease transmission. Transmission models may also be useful for short-term forecasting: when it is feasible to assume that key epidemiologic features will remain constant over time, such models can provide projections of natural transmission dynamics given the current state of an epidemic. Models cannot predict the future with certainty, but they can be helpful for scenario analysis by bounding the range of plausible future trajectories^8^. At the same time, simple models may have poor inferential and predictive performance if they fail to capture important features of disease transmission that may vary over time.

In early 2020, many countries, including the US, faced a public health crisis caused by the COVID-19 pandemic. In places like New York City and large cities in California, COVID-19 cases increased rapidly. In New York City, severely ill patients overwhelmed hospitals^9,10^ with death rates as high as 9% among confirmed cases and 32% among hospitalized patients^11^. Policymakers from the US regions first affected by the pandemic, Connecticut being one of them, were unprepared for its magnitude and severity. In the absence of effective pharmaceutical interventions, state and local governments turned to public health control measures that were last widely used during the 1918 influenza pandemic, such as social distancing and stay-at-home orders, to slow transmission of SARS-CoV-2. As transmission subsided, states began considering phased lifting of social distancing restrictions. Several urgent questions emerged: (1) How soon can interventions like school closures and stay-at-home orders be lifted? (2) How should public health interventions be implemented to minimize the risk of a resurgence? (3) What will be the effect of phased reopening plans on cases, hospitalizations, and deaths? Surveillance data on testing, case counts, hospitalizations, and deaths were useful in characterizing the dynamics of the initial wave, but policymakers needed predictive analytic tools to evaluate the current state of an epidemic and assess the risk of future resurgence.

A wide variety of predictive and explanatory models were developed during the early months of the COVID-19 pandemic. They included both mechanistic and phenomenological (statistical) models and were constructed for several related purposes. Many models sought to estimate basic epidemiologic parameters including the basic reproduction number ($R_0$), epidemic growth rate and doubling time, serial interval, case and infection fatality ratios, and case detection ratio^6,12–20^. Some models produced simulated future outcomes under hypothetical behavioral or interventional scenarios^1,4,21^. Other models had a retrospective inferential goal of estimating effects of past interventions including lockdown and other non-pharmaceutical measures^6,14,16,18,20^. Some early models did not calibrate parameters to data, and instead simulated from models parameterized using published estimates from early observational studies or other infections with similar properties^1,4,21^. Intervention effects were often included as constant model parameters or estimated by calibrating the model separately to pre- and post-intervention time periods^6,15,16^. In the early stages of the pandemic, these models helped demonstrate the dangers of unmitigated transmission and provided some evidence of the effectiveness of non-pharmaceutical interventions. However most of these models relied on publicly available data, often limited to official case counts, from the first wave of an epidemic^12–20^, and assumed constant transition rates (other than reduction in transmission following initial lockdown), and were therefore unable to adequately capture changing features of the COVID-19 burden, including behavioral changes, time-varying policy response, clinical management of the disease, or healthcare system dynamics^1,13–16,19,21^. Due to limitations of predictive performance of mechanistic models at early stages of epidemics when data are scarce^22^, some researchers used phenomenological models for the purposes of short- and medium-term forecasting^23–25^. The advantage of data-driven statistical models compared to mechanistic models is that they require fewer assumptions and do not suffer from identifiability problems in a way that many SEIR-type models may^22^. However, these models lack the ability to take advantage of the known features of infection transmission process and therefore are sometimes unable to offer insights into important features of past and future epidemic dynamics. They have limited use in scenario analyses that aim to assess potential effects of hypothetical interventions.

As the initial epidemic wave in Connecticut began to subside during the summer of 2020, local policymakers needed models that could answer specific questions about past and current infection dynamics, accommodate established epidemiologic features of disease transmission, permit prediction of outcomes under policy scenarios identified by stakeholders, and provide projections of policy-relevant outcomes under assumptions that could be understood by policymakers. Several nationwide forecasting tools were developed to provide state- or county-level projections, relying on data universally and publicly available across all locations^3,7,26^. These models employed assumptions applied universally across all locations and provided a limited set of outputs that were not always able to address the needs of local policymakers. These factors motivated the development of transmission models tailored to local context, the timing of intervention events and planned future policy changes, and data that were only available at the local level^27–31^.

In this paper, we present a county-structured model of SARS-CoV-2 transmission and COVID-19 disease progression in Connecticut. The model was developed and improved over the first year of the pandemic to support decision-making by Connecticut public health officials and policymakers^32^. This work is the result of continuous feedback from Connecticut public health officials, who provided detailed data on congregate and non-congregate testing, cases, and deaths, age-stratified cases, and hospitalization admissions and census – a feature that is absent from all nationwide analyses. We first describe the epidemic and public policy response in Connecticut, emphasizing the inferential questions articulated by public officials during development of the model. We then briefly describe the structure of the transmission model, its parameters, and data sources used for model calibration, with full details given in the Supplementary Material. We present results, including model predictions of hospitalizations and deaths on the dates of important decisions made by policymakers, estimates of the number of COVID-19 infections, case-detection ratio (CDR), cumulative incidence, effective reproduction number ($R_{\mathrm{eff}}$), infection fatality ratio (IFR), and other epidemiologic parameters. We conclude with a discussion of the limitations inherent in predicting uncertain epidemic trajectories using models, and outline lessons learned from one year of providing COVID-19 projections to support Connecticut policymakers.

### The COVID-19 epidemic and response in Connecticut

Connecticut (population 3.565 million) was among the US states most severely impacted by the first wave of COVID-19 epidemic^33^. On March 8th, 2020, the first Connecticut COVID-19 case was reported, followed by a rapid increase in case counts. In the first three weeks of the epidemic, the state reported over 2500 confirmed COVID-19 cases^34^. A similar rate of increase in hospitalizations followed, and on April 2, 2020, COVID-19 hospitalization census exceeded 1000. On March 17, Governor Ned Lamont ordered all in-person classes at K-12 schools canceled, and later extended the closure for the remainder of the 2019–2020 academic year^35–38^. The Governor issued a statewide “Stay Safe, Stay Home” order to take effect on March 23^39^. The order called on all nonessential businesses to cease in-person operations. Essential businesses could remain open with additional restrictions and guidelines to minimize close contact and risk of transmission. Evidence from mobile device data suggests that Connecticut residents reduced their mobility before the official lockdown order went in effect^40^.

The number of hospitalized COVID-19 patients in Connecticut peaked on April 21, 2020 and began a slow decline^34^. In early May, Governor Lamont issued plans and guidance for reopening, a process set to begin with “Phase 1” on May 20 when some businesses, mostly those operating outdoors, were allowed to reopen at 50% capacity^41^. Around the same time, we released a report that included COVID-19 transmission projections through August 31, 2020 under different scenarios of potential contact increase during the summer^32^. Phase 2 of reopening began on June 17th when indoor dining, libraries and religious services were allowed to reopen at reduced capacity^42^. Reopening was followed by scale-up in testing: the average daily number of polymerase chain reaction (PCR) tests increased from about 2,000 at the beginning of April to about 8,000 at the end of June^34^.

For most of summer 2020, case counts and hospitalizations in Connecticut remained low, even while large outbreaks were happening in many parts of the US^34^. The major state-level policy question during this time was whether and how to reopen primary, secondary, and college/university schooling in the fall. At the end of August, we developed model projections of infections, hospitalizations, and deaths for fall under different assumptions about the rate of close interpersonal contact associated with reopening of schools. These forecasts predicted increasing infections and a statewide resurgence during the fall 2020. In-person, remote, and hybrid education at all levels resumed in Connecticut in August and early September. Case counts and hospitalizations during fall 2020 increased slowly^34^, leading the Governor to implement Phase 3 reopening, permitting indoor businesses to operate at higher capacity, on October 8^43^. By early November, public health officials recognized a broad statewide epidemic resurgence. In response to rising case counts and fears of a substantial second wave, on November 6 Governor Lamont reverted to “Phase 2.1”, reducing permitted occupancy of indoor businesses and events^44^. In-person education at most public schools and all universities ended in mid-November before the Thanksgiving recess. Asymptomatic testing programs implemented by many universities were subsequently scaled down, resulting in a reduction in testing rates in Connecticut – making it difficult to interpret changes in the test positive proportion. Case counts in the second wave of the epidemic peaked in mid-December, plateaued for about a month and began a slow decline starting the second half of January 2021.

As of mid-March 2021, most schools and universities have reopened with a simultaneous substantial increase in close contact rates. Vaccine deployment in Connecticut began on December 14th, 2020 with residents of congregate settings being vaccinated first. As of March 1, 2021, 8.9% of Connecticut population received a full vaccination schedule and 16.5% received at least one dose of the vaccine with most vaccines being administered among residents of congregate settings and in the age group of 75 year old and above^34^.

## Methods

### Data sources

**Figure 1:**
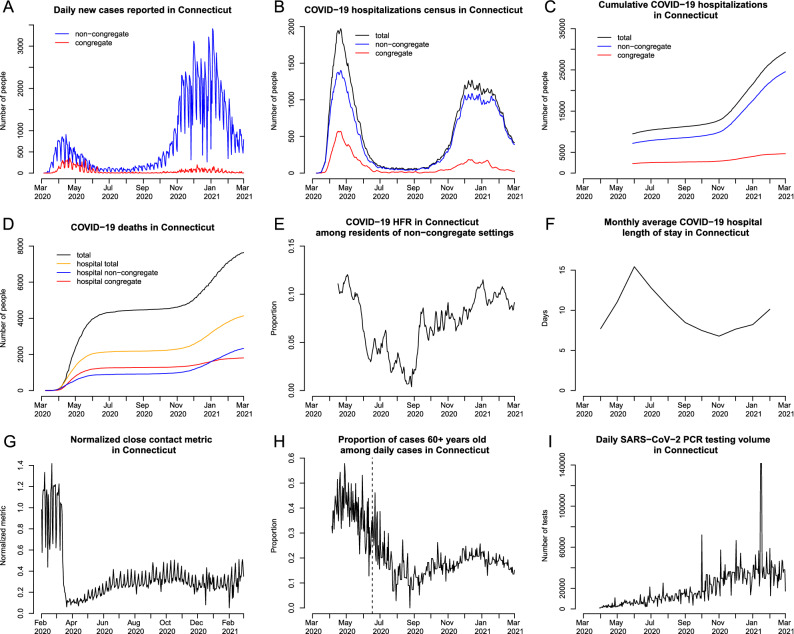
Observed and estimated data used in model calibration and approximation of time-varying model parameters. (**A**) daily new cases reported in Connecticut by the date of specimen collection among residents of non-congregate and congregate settings; (**B**) COVID-19 hospitalization census; (**C**) cumulative COVID-19 hospitalizations. In plots (**B**) and (**C**), the total number (black line) represents observed data, while non-congregate (blue) and congregate (red) lines represent estimates. (**D**) cumulative COVID-19 deaths in Connecticut; (**E**) hospital case fatality ratio (HFR) among hospitalized residents of non-congregate settings (estimated); (**F**) average length of hospital stay among COVID-19 patients by month; (**G**) normalized close interpersonal contact metric relative to the pre-epidemic period; (**H**) proportion of cases 60+ years old among daily COVID-19 cases (only data on the right of the dashed line is used in model parametrization); (**I**) daily PCR testing volume.

Our modeling approach relies on multiple data streams provided by the Connecticut Department of Public Health (CT DPH) and the Connecticut Hospital Association (CHA). Some of these datasets are publicly available, while others, including public health surveillance data, were obtained through a contract agreement between CT DPH and the Yale School of Public Health. Baseline non-institutionalized county-level populations and age demographics in Connecticut were obtained from the American Community Survey^45^. Figure 1 shows data series, described below, used in model parametrization and calibration.

#### Hospitalization, deaths, and hospital capacity

We obtained data on daily confirmed COVID-19 hospitalization census, cumulative hospitalizations, cumulative number of deaths among hospitalized patients, and daily total available hospital beds (including occupied) in Connecticut from CHA^46^. Hospitalization census and deaths time series were available since the epidemic onset in March 2020, while cumulative hospitalizations data were available starting May 29, 2020. Data on the total number of COVID-19 deaths are publicly available and were obtained from the Connecticut Open Data Portal^34^.

The transmission model aims to capture community spread of SARS-CoV-2, and therefore excludes transmission occurring in congregate settings like skilled nursing and assisted living facilities, or prisons. Similar to^6^, we excluded congregate settings, since transmission in small closed communities violates important modeling assumptions related to mixing patterns in the population. Available hospitalization data do not disaggregate by the patient’s place of residence at the time of diagnosis or hospitalization. According to CT DPH, as of October 30, 2020, about 73% of all deaths have occurred among residents of congregate settings, primarily nursing homes, emphasizing that the first wave of the epidemic was heavily dominated by transmission in this population. To address this issue, we estimated the time series of hospitalizations (census and cumulative) coming from non-congregate settings and used these estimated counts in the model calibration. We received data on daily COVID-19 death counts in hospitals disaggregated by the type of residence (congregate vs. non-congregate) at the time of diagnosis or hospitalization from CT DPH (Fig. 1D). Based on these data, we estimated the time-varying proportion of hospitalization census and cumulative hospitalizations coming from congregate and non-congregate settings. A detailed description of this process is provided in the Supplement. Plots B and C in Fig. 1 show estimates of these time series along with observed total numbers.

Data on estimated daily hospital admissions and deaths from non-congregate settings were used to estimate time-varying hospital case fatality ratio (HFR) and used as a time-varying parameter in the transmission model (Fig. 1E). Data on monthly average hospital length of stay among COVID-19 inpatients in Connecticut hospitals were provided by the CHA and used as a time-varying parameter in the transmission model (Fig. 1F).

#### COVID-19 tests, cases, and age distribution of cases

We assume that widespread testing shortens the time between infection and diagnosis and may therefore lead to shorter duration of transmissibility via isolation of infected individuals. We use daily PCR testing volume (Fig. 1I) to parameterize the time-varying duration of infectiousness among mildly symptomatic and asymptomatic cases. Data on daily PCR tests were obtained from the Connecticut Open Data Portal^34^.

We used the proportion of daily confirmed COVID-19 cases aged 60 years old and above to parameterize the dynamics of severe infections over time (Fig. 1H). Early in the epidemic, testing was not widely available and was primarily used to confirm severe cases that were more likely to be among older people. Therefore, in model parametrization, we assume a constant severe proportion early in the epidemic, and use these data to approximate changing severity proportion beyond the second phase of reopening, which started on June 17, 2020, when testing became widely available (vertical dashed line in Fig. 1H). Plot A in Fig. 1 shows reported case counts in Connecticut by residence type (congregate or non-congregate).

#### Close interpersonal contact

Close interpersonal contact (within six feet) is the main route for transmission of SARS-CoV-2^47^. Social distancing interventions implemented in Connecticut were intended to reduce the frequency of such contact. We therefore estimated the frequency of close interpersonal contact everywhere in Connecticut using mobile device geolocation data. The project, described separately in^48^, developed a novel probabilistic measure of contact and aggregated contact events at the town and state levels to describe the dynamics of close interpersonal contact. Figure 1G shows that statewide close contact in Connecticut dropped from its February 2020 baseline about one week prior to the Governor’s stay-at-home order, and rose slowly throughout the summer and fall.

### Compartmental model

**Figure 2:**
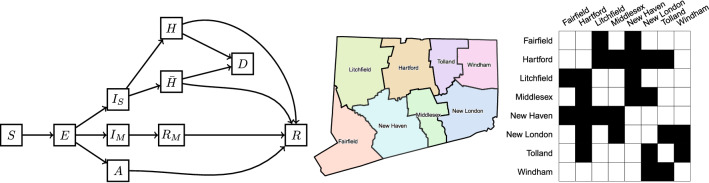
Schematic illustration of the model of SARS-CoV-2 transmission and COVID-19 disease progression; county map of Connecticut and county adjacency matrix. Individuals begin in the susceptible (*S*) compartment. Exposed individuals (*E*) may develop either asymptomatic (*A*), mild ($I_M$), or severe ($I_S$) infection. Asymptomatic and mild infections resolve without hospitalization and do not lead to death. Mild symptomatic cases self-isolate ($R_M$) shortly after development of symptoms, and transition to recovery (*R*) when infectiousness ceases. All severe cases require hospitalization (*H*) unless hospitalization capacity is exhausted, in which case they transition to ${\bar{H}}$ representing hospital overflow, then to recovery (*R*) or death (*D*). The model captures infection transmission in non-congregate settings, and excludes cases and deaths occurring in settings like nursing homes and prisons. It assumes a closed population without births and does not capture non-COVID-19 deaths. In the adjacency matrix, the black cells correspond to counties that are adjacent.

We developed a deterministic compartmental model of SARS-CoV-2 transmission and COVID-19 disease progression. The model is based on the SEIR (susceptible, exposed, infectious, removed) framework^49^, which we extended to accommodate geographical variation in Connecticut, hospital capacity, and distinctive features of COVID-19 disease. The model is implemented at the level of individual counties in Connecticut and assumes that most transmission occurs within a given county. A small proportion of contacts (1.5%) is allowed to happen between adjacent counties. Figure 2 shows a schematic representation of the transmission model structure within a single county, the county map of Connecticut^50^, and the county adjacency matrix.

We categorize infections as asymptomatic (*A*), mild symptomatic ($I_M$), and severe ($I_S$). Severe infections require hospitalization (*H*) and may lead to death (*D*). If hospitalization capacity is overwhelmed, severe cases in the community are denied hospitalization (${\bar{H}}$), and experience a higher probability of death compared to hospitalized cases. Mild symptomatic cases are assumed to self-isolate shortly after they develop symptoms ($R_M$) and remain isolated until they recover (*R*). The force of infection from hospitalized patients to unhospitalized susceptible individuals is assumed to be negligible due to infection control measures, including patient isolation and use of personal protective equipment by medical workers. In light of the long-lasting immunity conferred by natural SARS-CoV-2 infection^51,52^, we further assume that recovered individuals remain immune to reinfection for the duration of the study period. While reinfections are possible, they remained extremely rare during the time frame of this study and are therefore assumed to be negligible^52^. Let $N_i$ be the population size of county *i* and let $J_i$ be the set of counties adjacent to county *i*. Let $C^{(i)}$ represent hospitalization capacity in county *i*, which may vary over time. Transmission dynamics in county *i* are given by the following system of ordinary differential equations:

1 $$\begin{aligned} \begin{aligned} \frac{\ \text {d} S^{(i)}}{\ \text {d} t}&= -\beta S^{(i)} \left[ (1-k_n) \frac{ I_M^{(i)} + I_S^{(i)} + k_A A^{(i)}}{N_i} + \frac{k_n}{|J_i|} \sum _{j\in J_i} \frac{I_M^{(j)} + I_S^{(j)} + k_A A^{(j)}}{N_j} \right] \\ \frac{\ \text {d} E^{(i)}}{\ \text {d} t}&= \beta S^{(i)} \left[ (1-k_n) \frac{ I_M^{(i)} + I_S^{(i)} + k_A A^{(i)}}{N_i} + \frac{k_n}{|J_i|} \sum _{j\in J_i} \frac{I_M^{(j)} + I_S^{(j)} + k_A A^{(j)}}{N_j} \right] - \delta E^{(i)} \\ \frac{\ \text {d} A^{(i)}}{\ \text {d} t}&= q_A \delta E^{(i)} - \alpha _A A^{(i)} \\ \frac{\ \text {d} I_M^{(i)}}{\ \text {d} t}&= q_{I_M} \delta E^{(i)} - \alpha _{I_M} I_M^{(i)} \\ \frac{\ \text {d} R_M^{(i)}}{\ \text {d} t}&= \alpha _{I_M} I_M^{(i)} - \gamma _{R_M} R_M^{(i)} \\ \frac{\ \text {d} I_S^{(i)}}{\ \text {d} t}&= q_{I_S} \delta E^{(i)} - \alpha _{I_S} I_S^{(i)} \\ \frac{\ \text {d} H^{(i)}}{\ \text {d} t}&= (1 - \eta ^{(i)}) \alpha _{I_S} I_S^{(i)} - \gamma _H H^{(i)} \\ \frac{\ \text {d} {\bar{H}}^{(i)}}{\ \text {d} t}&= \eta ^{(i)} \alpha _{I_S} I_S^{(i)} - \gamma _{{\bar{H}}} {\bar{H}}^{(i)} \\ \frac{\ \text {d} D^{(i)}}{\ \text {d} t}&= \gamma _H m_H H^{(i)} + \gamma _{{\bar{H}}} m_{{\bar{H}}} {\bar{H}}^{(i)} \\ \frac{\ \text {d} R^{(i)}}{\ \text {d} t}&= \alpha _A A^{(i)} + \gamma _{R_M} R_M^{(i)} + \gamma _H (1-m_H) H^{(i)} + \gamma _{{\bar{H}}} (1 - m_{{\bar{H}}}) {\bar{H}}^{(i)},\\ \end{aligned} \end{aligned}$$

where $q_A + q_{I_M} + q_{I_S} = 1$. The function $\eta ^{(i)} = \left[ 1+\exp (0.5(C^{(i)}-H^{(i)})) \right] ^{-1}$ is a “soft” continuous hospitalization capacity overflow function. In practice, this implies that if hospitalization capacity is exceeded by 10 or more people, over 99% of new severe infections will be denied admission, meaning that the difference between the continuous and sharp thresholds is negligible. Table 1 lists definitions of model parameters, some of which are time-varying as explained in the next section. The analysis was performed using the R statistical computing environment^53,54^.

Our transmission model does not include effects of vaccination, which began on December 14th, 2020. Initial vaccine deployment in Connecticut prioritized residents of congregate settings and individuals in the age group 75 years old and above. According to the CDC, COVID-19 vaccines achieve their full effectiveness 14 days after the second dose in a 2-dose series or a single dose in a 1-dose series^55^. As of March 1, 2021, about 5% of non-congregate population in Connecticut were fully vaccinated. Given that some of these people may have already experienced SARS-CoV-2 infection, that the majority of them were older people who do not mix as much as younger working-age population, and that the proportion vaccinated is within the uncertainty bounds of the cumulative incidence, vaccine effects before March 1, 2021 are unlikely to have a substantial impact on model projections. At the same time, some of the vaccine effects, such as incidence and case fatality reduction among older people are captured in the dynamics of time-varying model parameters. As vaccination coverage among young and working-age individuals increases, it will become important to incorporate vaccine effects in the transmission model.

**Table 1:** Transmission model parameters.

Notation	Definition
$\beta$	Transmission parameter per susceptible–infectious pair
$\delta$	1 / Latency period (days$^{-1}$)
$q_A$, $q_{I_M}$, $q_{I_S}$	Proportions of infections that are asymptomatic, mild symptomatic, and severe, $q_A+q_{I_M}+q_{I_S}=1$
$\alpha _A$	1 / Duration of infectiousness among asymptomatic cases (days$^{-1}$)
$k_A$	Relative infectiousness of asymptomatic cases compared to symptomatic
$\alpha _{I_M}$	1 / Duration of infectiousness among mild symptomatic cases, time until isolation (days$^{-1}$)
$\gamma _{R_M}$	1 / Duration of isolation among mild symptomatic cases, remaining time to recovery (days$^{-1}$)
$\alpha _{I_S}$	1 / Duration of infectiousness among severe cases, time to hospitalization (days$^{-1}$)
$\gamma _{H}$	1 / Length of hospital stay (time until recovery or death) (days$^{-1}$)
$\gamma _{{\bar{H}}}$	1 / Remaining time until recovery or death among hospital overflow patients$^{-1}$ (days$^{-1}$)
$m_H$	Case fatality ratio among hospitalized cases (HFR)
$m_{{\bar{H}}}$	Case fatality ratio among hospital overflow patients
$k_n$	Proportion of all contacts that happen with individuals from adjacent counties (as opposed to within the county)
*C*	Hospitalization capacity, may be constant or vary over time representing capacity increase intervention
$E_0$	Number of exposed individuals statewide at the time of epidemic onset (initial condition, see Supplementary Material for details)

### Time-varying model parameters

#### Transmission rate $\beta$

Social distancing practices may reduce the value of the transmission rate $\beta$. We use data on close interpersonal contact in Connecticut for the entire duration of the modeling period (Fig. 1G)^48^, and assume the following functional form for the transmission rate:

$$\begin{aligned} \beta (t) = \beta _0 M_{\mathrm{contact}}(t) \exp [B(t)] , \end{aligned}$$

where $M_{\mathrm{contact}}(t)$ is a smoothed normalized measure of close interpersonal contact at time *t* relative to the pre-epidemic level (February 1st–March 12th, 2020), and $\exp [B(t)]$ is a function that approximates residual changes in transmission parameter $\beta$ that are not explained by changes in close contact and other time-varying parameters. Here, *B*(*t*) is a smooth function obtained by applying spline smoothing on a piecewise linear function $B^*(t)$ defined as follows:

$$\begin{aligned} B^*(t) = \epsilon _{w(t)} + \frac{\epsilon _{w(t)+1} - \epsilon _{w(t)}}{14} (t - t_{w(t)}); \;\;\; t \in [t_{w(t)} ; t_{w(t)+1}), \;\; w(t) = \{ 0,1,2,\ldots \}, \end{aligned}$$

where *w*(*t*) indexes bi-weekly knots starting at time ${\tilde{t}}$, and $t_{w(t)} = \{{\tilde{t}}, {\tilde{t}}+14, {\tilde{t}}+28, \ldots \}$ is the day corresponding to the beginning of bi-weekly interval *w*(*t*). We model the vector of random effects $\varvec{\epsilon }$ using a random walk of order one and calibrate it to observed data (see Supplement for details):

$$\begin{aligned} \epsilon _0 = 0, \;\;\;\; \epsilon _{w(t)+1}|\epsilon _{w(t)} \sim {\mathcal {N}}(\epsilon _{w(t)}, \sigma _\epsilon ^2). \end{aligned}$$

For the hyperparameter $\sigma _\epsilon ^2$, we use Inverse-Gamma$(a_\epsilon , b_\epsilon )$ prior with a shape parameter $a_\epsilon = 2.5$ and a rate parameter $b_\epsilon = 0.1$. The Supplement shows plots of functions $M_{\mathrm{contact}}(t)$ and *B*(*t*). The function *B*(*t*) is also used to set $\beta (t)$ in the future to test scenarios and potential intervention effects.

#### Rates of isolation and recovery: $\alpha _{I_M}$*and*$\alpha _A$

Widespread testing and contact tracing efforts can potentially reduce duration of infectiousness. While there is no information about the effectiveness of specific testing efforts implemented in Connecticut, the model accommodates the possibility of such reduction as a function of daily testing volume:

$$\begin{aligned} \alpha (t) = \alpha _0 (1 + M_{\mathrm{testing}}(t) \tau ) , \end{aligned}$$

where $\tau$ is the size of testing effect per unit increase in testing volume measure $M_{\mathrm{testing}}(t)$ modeled as:

$$\begin{aligned} M_{\mathrm{testing}}(t) = {\left\{ \begin{array}{ll} \log \left( v_{\mathrm{testing}}(t) \right) - \log \left( v_{\mathrm{testing}}(t^*) \right) , &{} t > t^* \text { and } v_{\mathrm{testing}}(t) \ge v_{\mathrm{testing}}(t^*) \\ 0, &{} \text {otherwise.} \end{array}\right. } \end{aligned}$$

$v_{\mathrm{testing}}(t)$ is a spline-smoothed measure of testing volume at time *t*. Testing efforts early in the epidemic were primarily used to confirm severe and highly symptomatic infections, and were unlikely to have any appreciable impact on overall duration of infectiousness. Early response daily testing volume is denoted by $v_{\mathrm{testing}}(t^*)$. The Supplement shows the plot of function $M_{\mathrm{testing}}(t)$. This approach is used to model time-varying rates $\alpha _{I_M}(t)$ and $\alpha _A(t)$ with $\tau _{I_M} = \tau$ and $\tau _A = 0.5 \tau$. The rate $\alpha _{I_S}$ is assumed to remain constant over time.

#### Severe fraction $q_{I_S}$

The probability of severe infection increases with age^56^. Age distribution of confirmed cases in the US has shifted toward younger people in the summer compared to spring^57^. We model the time-varying proportion of infections that are severe as:

$$\begin{aligned} q_{I_S}(t) = q_{I_S,0} M_{\mathrm{severity}}(t) , \end{aligned}$$

where measure of severity $M_{\mathrm{severity}}(t)$ is a normalized spline-smoothed proportion of cases 60+ years old among all cases detected at time *t* relative to a baseline level. Since testing availability affects this proportion, we assume that $M_{\mathrm{severity}}(t)=1$ for all $t < t^*$, where $t^*$ denotes the time when testing became widely available. The Supplement shows the plot of function $M_{\mathrm{severity}}(t)$. Since $q_A+q_{I_M}+q_{I_S}=1$, we also model $q_A$ and $q_{I_M}$ as functions of time.

#### Rate of hospital discharge $\gamma _H$

We estimate the time-varying rate of hospital discharge $\gamma _H(t)$ (including deaths and alive discharges) as a reciprocal of the average length of hospital stay at time *t*, which is approximated using a spline-smoothed monthly averages of this quantity and is provided in the Supplement.

#### Hospital case fatality ratio $m_H$

Overall and hospital case fatality ratios may vary over time for various reasons. We model HFR as:

$$\begin{aligned} m_H(t) = m_{H,0} M_{\mathrm{HFR}}(t) , \end{aligned}$$

where $M_{\mathrm{HFR}}(t)$ is a normalized spline-smoothed HFR at time *t* relative to the baseline $\text {HFR} = m_{H,0}$. HFR at time *t* is estimated as a ratio of hospital deaths at time *t* to hospital admissions at time $(t-\text {HLOS}(t))$, where $\text {HLOS}(t)$ is an average hospital length of stay at time *t*. The Supplement shows the plot of function $M_{\mathrm{HFR}}(t)$.

### Model calibration and Bayesian posterior inference

We calibrate the posterior distribution of model parameters to estimated statewide hospitalizations and hospital deaths coming from non-congregate settings using a Bayesian approach. Sampling from the joint posterior distribution of calibrated model parameters is performed using Markov Chain Monte Carlo. The Supplement provides detailed description of the data likelihood, calibration approach, sampling algorithm, implementation, and results, including convergence and estimated joint posterior distribution of model parameters, as well as description of prior distributions along with data sources. To generate model projections, we sample from the joint posterior distribution of estimated parameters, simulate transmission dynamics for a given combination of parameters, and compute pointwise averages (means or medians) and posterior predictive intervals for each time point.

### Estimates of epidemiologic parameters

We provide model-based estimates of the following epidemiologic parameters: basic ($R_0$) and effective ($R_{\mathrm{eff}}$) reproduction number, instantaneous and cumulative case detection ratio (CDR), infection hospitalization ratio (IHR), infection fatality ratio (IFR), and hospital case fatality ratio (HFR). Definitions of these parameters and model-based estimation details are provided in the Supplement.

### Ethics statement

This analysis is based on aggregated data and does not use patient-level data with personal identifiers.

## Results

### Estimates of epidemiologic features

**Figure 3:**
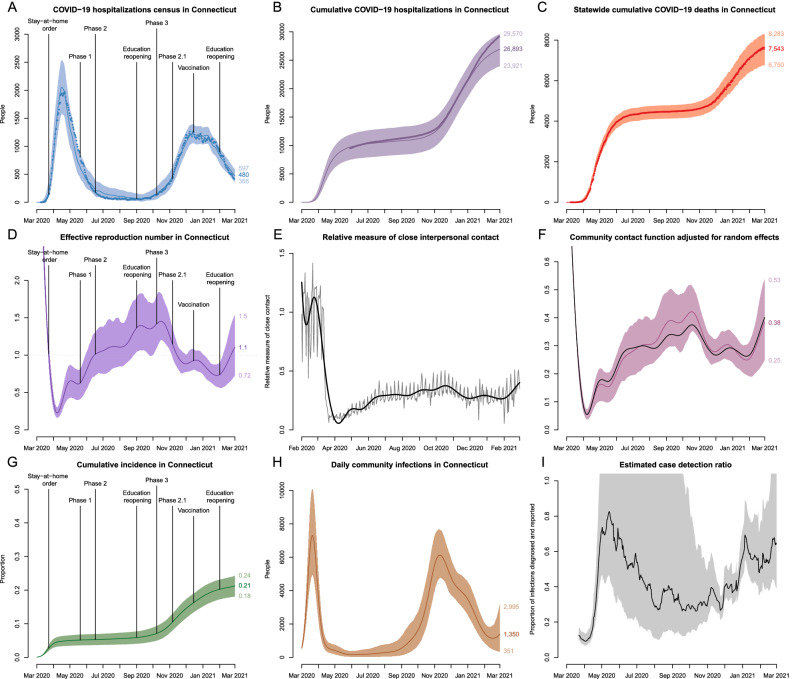
Model fit to observed data and estimates of epidemiologic features of SARS-CoV-2 transmission in Connecticut. Top row shows calibration results for: (**A**) observed COVID-19 hospitalizations census, (**B**) cumulative hospitalizations and (**C**) cumulative deaths in Connecticut. Observed time series are shown as points and correspond to total hospitalizations and deaths among all Connecticut residents. The model is calibrated to estimated data series coming from non-congregate settings, and model projections are adjusted by the estimated difference to reflect the totals for congregate and non-congregate settings. (**D**) effective reproduction number; (**E**) normalized measure of close contact relative to the pre-epidemic period along with the spline approximation (thick solid line); (**F**) contact function adjusted for estimated random effects that capture residual variation in transmission that is not explained by dynamics of close contact and other time-varying parameters. For comparison, black line in plot (**F**) shows smoothed normalized close contact metric unadjusted for random effects. (**G**) cumulative incidence of SARS-CoV-2 infection; (**H**) daily new infections; (**I**): estimated case detection ratio in non-congregate settings in Connecticut. Solid lines represent model-projected means and shaded regions represent 95% posterior predictive intervals. Some of the plots are overlaid with the key intervention dates (lockdown and phased reopening), as well as important event dates, including reopening of schools and universities and beginning of the vaccination campaign.

Figure 3 shows the results of model calibration along with estimates of important epidemiologic parameters for the period between March 1, 2020–March 1, 2021. Plots of hospitalizations, effective reproduction number ($R_{\mathrm{eff}}$), and cumulative incidence are overlaid with the dates of Governor’s interventions, reopening of schools and universities in the fall and winter, and the starting date of vaccination campaign. Model fit to observed dynamics of hospitalizations and deaths shows that data points track with mean projections and largely fall within uncertainty intervals.

We estimate that $R_{\mathrm{eff}}$ dropped substantially in mid-March and remained below one through mid-June. For the rest of the summer, mean estimated $R_{\mathrm{eff}}$ was slightly above one consistent with low numbers of case counts and hospitalizations in the summer. A major increase of $R_{\mathrm{eff}}$ started in mid-August and continued with the reopening of schools and colleges. It reached a maximum mean value of 1.45 by mid-October, followed by a slow decline through the rest of the year. A second increase in $R_{\mathrm{eff}}$ started at the end of January 2021. At this time, vaccine coverage that was targeted at people over 65 years old and congregate settings residents was insufficient to counteract increase in close contact likely associated with reopening of schools and universities for the spring semester. The dynamics of $R_{\mathrm{eff}}$ follows closely the dynamics of close contact. Figure 3F shows that the estimated dynamics of transmission parameter captured by a community contact function (measure of close contact adjusted for estimated random effects) exhibits small deviations from the measure of close contact. Our estimate of $R_0$ is 4.7 (95%CI: 4.3–5.1), consistent with estimates reported elsewhere^58,59^. However, this estimate depends on assumptions about initial conditions and is not identifiable from the shape of early exponential increase alone.

We estimate that cumulative incidence at the beginning of June 2020 was 5.2% (95% CI: 3.6–6.8%) consistent with the results of community-based seroprevalence surveys conducted in Connecticut between April and June^60,61^ and other local modeling efforts that included Connecticut^62^. We estimate that as of March 1, 2021, cumulative incidence in Connecticut was about 21% (95% CI: 18–24%), which implies a cumulative case detection ratio of 36% (95% CI: 26–54%). Our estimates suggest that the case detection ratio varied substantially over time in a way that is not explained by the volume of PCR testing alone (Fig. 3I). An increase up to 80% in mid-May may be due to delayed testing, including postmortem diagnosis of first epidemic wave cases. The second spike in estimated case detection ratio in early January may be a consequence of testing and reporting disruptions related to the Christmas and New Year holidays.

We estimate the cumulative infection fatality ratio (IFR) to be 1.06% (95% CI: 0.93–1.24%) and the cumulative infection hospitalization ratio (IHR) to be 4.1% (95% CI: 3.6–4.7%). These estimates are based on data from all recorded deceased and hospitalized individuals, including those residing in congregate settings. Estimated IFR and IHR among residents of non-congregate settings are 0.43% (95% CI: 0.38–0.50%) and 3.4% (95% CI: 3.0–4.0%) respectively, which are somewhat lower than previously estimated in Connecticut^63^, but are consistent with studies conducted elsewhere^6,14,56,64^.

### Predictive performance of the model

**Figure 4:**
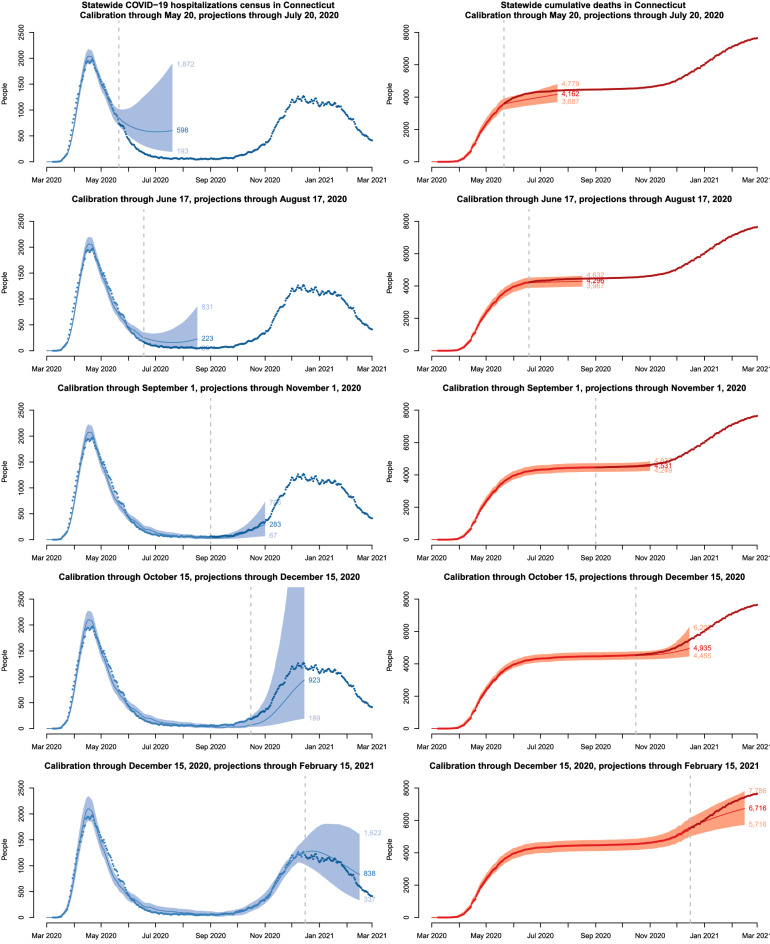
Posterior predictive performance of the transmission model calibrated using data up to the dashed line shown in each plot and projected forward for a period of two months. Solid lines represent model-projected means and shaded regions represent 90% posterior predictive intervals. Observed data are shown as points; lighter color points correspond to the data used in calibration.

We illustrate predictive performance of the model by calibrating it to data up to several time points and producing projections of hospitalizations and deaths for the next two months. Projections into the future are simulated by propagating the latest available values of time-varying parameters into the future. A forecasting time horizon of two months is shown. Longer-term projections were less useful due to anticipated changes in policy, public behavior, and time-varying parameters. The time points were selected based on important events, such as reopening phases, as well as distinct stages of the epidemic. Figure 4 shows calibrations results, projections and actual data over the prediction period for the following calibration cut-off dates: May 20, 2020 (Phase 1 of reopening), June 17, 2020 (Phase 2 of reopening), September 1, 2020 (reopening of schools and colleges), October 15, 2020 (early indications of resurgence), and December 15, 2020 (early indications of the curve flattening during the second epidemic wave).

These results show that projections become more accurate as data accrue, and that uncertainty intervals are generally tighter during periods of epidemic decline, when the upper bound of uncertainty interval for projected $R_{\mathrm{eff}}$ is below one. In early stages, our projections predicted higher hospitalization census compared to what was observed during summer 2020. The main reason for this mismatch is a sharp decline in severe infections proportion that was observed after the initial epidemic wave subsided, as well as the decline in the average length of hospital stay among hospitalized COVID-19 patients (plots H and F in Fig. 1).

## Discussion: transmission modeling in the COVID-19 pandemic

As COVID-19 pandemic emerged, policymakers in many parts of the US and across the globe had to make quick decisions under high uncertainty. Access to reliable information about both the current state of the local epidemic and likely future outcomes is an important foundation to support policymakers in this process. Elected officials and public health agencies already have access to near real-time information about COVID-19 testing, case counts, hospitalizations, and deaths. However, these data may not provide timely insight into the current and future dynamics of COVID-19 transmission. Mathematical modeling of infectious diseases offers one way to address these questions, but is subject to limitations that may be difficult to communicate to policymakers. During our year-long collaboration with CT DPH, we learned several lessons in the process of developing, deploying, calibrating, revising, and communicating the outputs of COVID-19 transmission model to policymakers in Connecticut.

### Transmission dynamics in congregate settings may bias model-based estimates

Observable features of the initial epidemic wave in Connecticut - hospitalizations and deaths—were dominated by residents of skilled nursing and assisted living facilities^34^. In our initial efforts to model outcomes under the state’s stated reopening plans in May of 2020, we did not isolate transmission in congregate settings and treated all hospitalizations and deaths as those arising from a homogeneous mixing process within Connecticut population^32^. Transmission dynamics in congregate settings among higher risk individuals violate the homogeneous mixing assumption underlying compartmental modeling approaches. Merging cases, hospitalizations and deaths from congregate and non-congregate settings may bias estimates of $R_{\mathrm{eff}}$ and result in over-estimation of cumulative incidence, transmission potential, and infection fatality ratio. Recognizing this, we modified the model to only include residents of non-congregate settings.

### Detailed local data are necessary to capture time-varying epidemic features

Similar to many early transmission models that only used data from the first epidemic wave^13–16,19^, the first version of our model did not incorporate any temporal variation in the input parameters and included constant effects of school closure and lockdown^32^. However, as the data accrued, constant parameter values failed to achieve a good fit to the observed data. Our collaboration with CT DPH allowed us access to detailed local data informing time-varying model parameters. Incorporating time trends in important epidemic features like close interpersonal contact, risk profile of incident cases, hospital length of stay, and hospital case fatality ratio substantially improved model fit to observed data and its predictive performance. One of the limitations of our model is that it does not incorporate vaccination effects. However, during the modeling period ending on March 1, 2021 these effects are likely negligible in non-congregate residents due to low overall coverage that is primarily driven by an older age group, and are partially captured in the dynamics of time-varying parameters. As vaccination coverage increases, it will be important to include vaccination effects in the transmission model.

### Anticipated changes in time-varying epidemic features warrant scenario analysis

The most substantial changes in time-varying model parameters occurred as the first epidemic wave began to subside in early summer 2020. Figure 4 illustrates modest predictive performance of our model during early stages. In this example, future projections are made by propagating the last observed value of time-varying model parameters into the future. When substantial changes in epidemic features are anticipated in the future, scenario analysis may offer a better way to represent the true uncertainty.

### Close interpersonal contact drives transmission

One of the most important data sources that substantially improved our model performance is a measure of close interpersonal contact based on mobile device data. This is a novel metric, whose dynamics exhibit different behavior from that observed in several publicly available mobility metrics, such as maximum distance traveled or time spent away from home, which mostly returned to their baseline values by mid-summer 2020^48^. Combined with other time-varying parameters, the close contact metric captured most of the variation in transmission over time. While our approach to use random effects to capture residual variation in transmission dynamics proved feasible and useful, the shape of the resulting contact function exhibits small deviations from the dynamics of close interpersonal contact (Fig. 3F).

### Timing of interventions may not always reflect timing of behavioral changes

Public perception of risk appears to be an important time-dependent confounder that is hard to measure or predict. Behavior changes that drive transmission, such as social distancing or mask wearing, may not coincide in time with respective interventions. In Connecticut, close interpersonal contact dropped substantially before the lockdown order went in effect and started rebounding before it was lifted. Many transmission models^6,15,16^, including the early version of our model^32^ assume constant intervention effects that modify a given set of parameters at the time of their enactment. Measuring critical features of transmission directly improves model-based projections and offers insights into the estimation of intervention effects.

### Case counts may be an unreliable proxy for infections

Policymakers often rely on case counts and test positive proportion as direct measures of infection incidence. Even though dynamics of detected cases may be a reasonable qualitative indicator of disease incidence, we found that its usefulness for modeling purposes is limited. It is widely recognized that detected case counts depend on the underlying infection incidence and testing volume^28,65^, however we found that changes in testing strategies and human behavior may lead to non-monotonic relationship between testing volume and case detection rate. Since case counts dynamics are usually widely available across geographic locations, and are often among a few data sources easily accessible by researchers, many nationwide models rely on case counts, possibly adjusting for testing volume^7,26^. Our experience shows that this approach may be misleading. We therefore chose to rely on hospitalizations and death data for model calibration as these epidemic features are less susceptible to unmeasured time-varying confounding.

### Incorporating diverse data sources improves credibility of model-based inferences

Our modeling projections have been one of the many analytic products informing policy response in Connecticut along with surveillance data on case counts, testing volume and test positive proportion, trends in emergency department visits presenting symptoms of COVID-like illness, outbreak investigations, wastewater monitoring^33^, and trends in close interpersonal contact^48^ among others. For a variety of reasons, it can be difficult for public health decision-makers to enact policy responses based on predictions about the future from transmission models. However, when model projections tell a cohesive story in combination with other analytic products, they may fill in missing pieces and offer insights into the current and future epidemic dynamics.

### Conclusions and limitations

Our year-long effort to provide policymakers with predictions of COVID-19 dynamics in Connecticut showed that standard SEIR-type transmission models work best in large well-mixing populations with constant transmission rates and without major external shocks, like interventions or importation of infections. In order to make valid inferences, it is necessary to incorporate temporal changes in model parameters reflecting interventions, human behavior, heterogeneity in mixing patterns, and measurement errors. Given geographic heterogeneity in the dynamics of these features, models that incorporate contextual information and local data are needed to support local policymakers. While modeling approach described in this paper captures many important features of epidemic dynamics that are often omitted in simpler SEIR-type models, our model could be extended to reflect more granular geographic variation, age structure and contact patterns between different age groups, vaccination effects, and other time-varying features of COVID-19 epidemic. When it works best, modeling provides heuristics, guidance, and what-if scenarios for the future offering insights that are otherwise unavailable.

## Supplementary information

Supplementary Information.

## Data Availability

Aggregated data used in this analysis and the model code are available from https://github.com/fcrawford/covid19_ct.

## References

[1] Ferguson, N. M. *et al.* Report 9: Impact of non-pharmaceutical interventions (NPIs) to reduce COVID-19 mortality and healthcare demand (2020). https://www.imperial.ac.uk/media/imperial-college/medicine/sph/ide/gida-fellowships/Imperial-College-COVID19-NPI-modelling-16-03-2020.pdf. 10.1007/s11538-020-00726-x PMC7140590 32270376

[2] Adam, D. Special report: The simulations driving the world’s response to COVID-19. *Nature* **580**, 316 (2020). 10.1038/d41586-020-01003-6 32242115

[3] Centers for Disease Control and Prevention. COVID-19 Mathematical Modeling. https://www.cdc.gov/coronavirus/2019-ncov/covid-data/mathematical-modeling.html (2020).

[4] Kissler SM, Tedijanto C, Goldstein E, Grad YH, Lipsitch M (2020). Projecting the transmission dynamics of SARS-CoV-2 through the postpandemic period. Science.

[5] Flaxman S (2020). Estimating the effects of non-pharmaceutical interventions on COVID-19 in Europe. Nature.

[6] Salje H (2020). Estimating the burden of SARS-CoV-2 in France. Science.

[7] IHME COVID-19 Forecasting Team. Modeling COVID-19 scenarios for the United States. *Nature Medicine* **27**, 94–105 (2021). 10.1038/s41591-020-1132-9 PMC7806509 33097835

[8] Holmdahl I, Buckee C (2020). Wrong but useful: what COVID-19 epidemiologic models can and cannot tell us. N. Engl. J. Med..

[9] Tanne JH (2020). COVID-19: New York City deaths pass 1000 as Trump tells Americans to distance for 30 days. BMJ.

[10] Uppal A (2020). Critical care and emergency department response at the epicenter of the COVID-19 pandemic. Health Affairs.

[11] Thompson CN (2020). COVID-19 outbreak: New York City, February 29-June 1, 2020. Morbidity Mortality Weekly Rep..

[12] Li Q (2020). Early transmission dynamics in Wuhan, China, of novel coronavirus-infected pneumonia. N. Engl. J. Med..

[13] Wu JT, Leung K, Leung GM (2020). Nowcasting and forecasting the potential domestic and international spread of the 2019-nCoV outbreak originating in Wuhan, China: a modelling study. Lancet.

[14] Wu JT (2020). Estimating clinical severity of COVID-19 from the transmission dynamics in Wuhan. China. Nature Med..

[15] Li R (2020). Substantial undocumented infection facilitates the rapid dissemination of novel coronavirus (SARS-CoV2). Science.

[16] Tian H (2020). An investigation of transmission control measures during the first 50 days of the COVID-19 epidemic in China. Science.

[17] Kucharski AJ (2020). Early dynamics of transmission and control of COVID-19: a mathematical modelling study. Lancet Infect. Dis..

[18] Leung K, Wu JT, Liu D, Leung GM (2020). First-wave COVID-19 transmissibility and severity in China outside Hubei after control measures, and second-wave scenario planning: a modelling impact assessment. Lancet.

[19] Hauser A (2020). Estimation of SARS-CoV-2 mortality during the early stages of an epidemic: a modeling study in Hubei, China, and six regions in Europe. PLoS Med..

[20] Dehning J (2020). Inferring change points in the spread of COVID-19 reveals the effectiveness of interventions. Science.

[21] Prem K (2020). The effect of control strategies to reduce social mixing on outcomes of the COVID-19 epidemic in Wuhan, China: a modelling study. Lancet Public Health.

[22] Roda WC, Varughese MB, Han D, Li MY (2020). Why is it difficult to accurately predict the COVID-19 epidemic?. Infect. Dis. Model..

[23] Murray, C. J. Forecasting COVID-19 impact on hospital bed-days, ICU-days, ventilator-days and deaths by US state in the next 4 months. *MedRxiv* (2020). https://www.medrxiv.org/content/10.1101/2020.03.27.20043752v1.

[24] Roosa K (2020). Real-time forecasts of the COVID-19 epidemic in China from February 5th to February 24th, 2020. Infect. Dis. Model..

[25] Osthus, D. Los Alamos National Laboratory COVID-19 cases and deaths forecasts. https://covid-19.bsvgateway.org (2020).

[26] Unwin HJT (2020). State-level tracking of COVID-19 in the United States. Nat. Commun..

[27] Aleta A (2020). Modelling the impact of testing, contact tracing and household quarantine on second waves of COVID-19. Nat. Hum. Behav..

[28] Fintzi, J. *et al.* Using multiple data streams to estimate and forecast SARS-CoV-2 transmission dynamics, with application to the virus spread in Orange County, California. *arXiv preprint* arXiv:2009.02654 (2020).

[29] Bracis C (2021). Widespread testing, case isolation and contact tracing may allow safe school reopening with continued moderate physical distancing: a modeling analysis of King County, WA data. Infect. Dis. Model..

[30] Tran, T. N.-A. *et al.* Optimal SARS-CoV-2 vaccine allocation using real-time seroprevalence estimates in Rhode Island and Massachusetts. *medRxiv* (2021). https://www.medrxiv.org/content/10.1101/2021.01.12.21249694v1. 10.1186/s12916-021-02038-w PMC8275456 34253200

[31] Kaplan EH (2020). COVID-19 scratch models to support local decisions. Manuf. Serv. Oper. Manag..

[32] Crawford, F. W., Li, Z. R. & Morozova, O. COVID-19 projections for reopening Connecticut. *medRxiv* (2020). https://www.medrxiv.org/content/early/2020/06/19/2020.06.16.20126425.

[33] Peccia J (2020). Measurement of SARS-CoV-2 RNA in wastewater tracks community infection dynamics. Nat. Biotechnol..

[34] State of Connecticut Office of Policy and Management. Connecticut Open Data: COVID-19 Data Resources. https://data.ct.gov/stories/s/COVID-19-data/wa3g-tfvc/. Accessed 29 March 2021.

[35] Lamont, N. Executive Order No. 7C: Protection of public health and safety during COVID-19 pandemic and response—further suspension or modification of statutes (March 15, 2020). https://portal.ct.gov/-/media/Office-of-the-Governor/Executive-Orders/Lamont-Executive-Orders/Executive-Order-No-7C.pdf.

[36] Lamont, N. Executive Order No. 7L: Protection of public health and safety during COVID-19 pandemic and response - extension of school cancellation, municipal retiree reemployment, open fishing season and additional public health measures (2020). https://portal.ct.gov/-/media/Office-of-the-Governor/Executive-Orders/Lamont-Executive-Orders/Executive-Order-No-7L.pdf.

[37] Lamont, N. Executive Order No. 7X: Protection of public health and safety during COVID-19 pandemic and response—renter protections, extended class cancellation and other safety measures, educator certification, food trucks for truckers (2020). https://portal.ct.gov/-/media/Office-of-the-Governor/Executive-Orders/Lamont-Executive-Orders/Executive-Order-No-7X.pdf.

[38] Lamont, N. Executive Order No 7II: Protection of public health and safety during COVID-19 pandemic and response—extension of school cancellation, home health care coverage, and food assistance measures (2020). https://portal.ct.gov/-/media/Office-of-the-Governor/Executive-Orders/Lamont-Executive-Orders/Executive-Order-No-7II.pdf.

[39] Lamont, N. Executive Order No. 7H: Protection of public health and safety during COVID-19 pandemic and response—restrictions on workplaces for non-essential businesses, coordinated response effort (March 20, 2020). https://portal.ct.gov/-/media/Office-of-the-Governor/Executive-Orders/Lamont-Executive-Orders/Executive-Order-No-7H.pdf.

[40] Centers for Disease Control and Prevention. COVID Data Tracker: Explore human mobility and COVID-19 transmission in your local area. https://covid.cdc.gov/covid-data-tracker/#mobility. Accessed: 2021-03-29.

[41] Lamont, N. Governor Lamont releases rules for businesses under First Phase of Connecticut’s reopening plans amid COVID-19. State of Connecticut Press Release (2020). https://portal.ct.gov/Office-of-the-Governor/News/Press-Releases/2020/05-2020/Governor-Lamont-Releases-Rules-for-Businesses-Under-First-Phase-of-Reopening-Plans.

[42] Lamont, N. Governor Lamont releases business documents for Phase 2 reopening on June 17. State of Connecticut Press Release (2020). https://portal.ct.gov/Office-of-the-Governor/News/Press-Releases/2020/06-2020/Governor-Lamont-Releases-Business-Documents-for-Phase-2-Reopening-on-June-17.

[43] Lamont, N. Governor Lamont announces Connecticut moves toward Phase 3 reopening on October 8. State of Connecticut Press Release (2020). https://portal.ct.gov/Office-of-the-Governor/News/Press-Releases/2020/09-2020/Governor-Lamont-Announces-Connecticut-Moves-Toward-Phase-3-Reopening-on-October-8.

[44] Lamont, N. Governor Lamont provides update on Connecticut’s coronavirus response efforts. State of Connecticut Press Release (2020). https://portal.ct.gov/Office-of-the-Governor/News/Press-Releases/2020/11-2020/Governor-Lamont-Coronavirus-Update-November-2.

[45] United States Census Bureau. *American Community Survey (ACS)* (2020). https://www.census.gov/programs-surveys/acs.

[46] Connecticut Hospital Association. https://cthosp.org/.

[47] Centers for Disease Control and Prevention. Scientific Brief: SARS-CoV-2 and Potential Airborne Transmission. https://www.cdc.gov/coronavirus/2019-ncov/more/scientific-brief-sars-cov-2.html (2020). 34009775

[48] Crawford, F. W. *et al.* Impact of close interpersonal contact on COVID-19 incidence: evidence from one year of mobile device data. *medRxiv* (2021). https://www.medrxiv.org/content/10.1101/2021.03.10.21253282v1. 10.1126/sciadv.abi5499 PMC8741180 34995121

[49] Keeling MJ, Rohani P (2011). Modeling Infectious Diseases in Humans and Animals.

[50] United States Census Bureau. 2010 cartographic boundary file, current block group for Connecticut. Data retrieved from http://magic.lib.uconn.edu/connecticut_data.html (2010). Accessed 14 April 2020.

[51] Radbruch A, Chang H-D (2021). A long-term perspective on immunity to COVID. Nature.

[52] Wadman M (2021). Having SARS-CoV-2 once confers much greater immunity than a vaccine—but vaccination remains vital. Science.

[53] R Core Team. *R: A Language and Environment for Statistical Computing*. R Foundation for Statistical Computing, Vienna, Austria (2020). https://www.R-project.org/.

[54] Soetaert K, Petzoldt T, Setzer RW (2010). Solving differential equations in R: Package deSolve. J. Stat. Softw..

[55] Centers for Disease Control and Prevention. COVID-19: When you’ve been fully vaccinated. https://www.cdc.gov/coronavirus/2019-ncov/vaccines/fully-vaccinated.html (2021). Accessed 05 April 2021.

[56] Verity R (2020). Estimates of the severity of coronavirus disease 2019: a model-based analysis. Lancet Infect. Dis..

[57] Boehmer TK (2020). Changing age distribution of the COVID-19 pandemic—United States, May–August 2020. Morbidity Mortality Weekly Rep..

[58] Katul GG, Mrad A, Bonetti S, Manoli G, Parolari AJ (2020). Global convergence of COVID-19 basic reproduction number and estimation from early-time SIR dynamics. PLoS ONE.

[59] Sanche S (2020). High contagiousness and rapid spread of severe acute respiratory syndrome coronavirus 2. Emerg. Infect. Dis..

[60] Havers FP (2020). Seroprevalence of antibodies to SARS-CoV-2 in 10 sites in the United States, March 23–May 12, 2020. JAMA Internal Med..

[61] Mahajan S (2021). Seroprevalence of SARS-CoV-2-specific IgG antibodies among adults living in Connecticut: post-infection prevalence (PIP) study. Am. J. Med..

[62] Boni, M. F. SARS-CoV-2 attack rates for Connecticut. https://twitter.com/maciekboni/status/1371847662077632513. Accessed 16 March 2021.

[63] Mahajan S (2021). SARS-CoV-2 infection hospitalization rate and infection fatality rate among the non-congregate population in Connecticut. Am. J. Med..

[64] Waltenburg MA (2020). Update: COVID-19 among workers in meat and poultry processing facilities—United States, April–May 2020. Morbidity Mortality Weekly Rep..

[65] Pitzer VE (2021). The impact of changes in diagnostic testing practices on estimates of COVID-19 transmission in the United States. Am. J. Epidemiol..

