# Supplementary material for: One year of modeling and forecasting COVID-19 transmission to support policymakers in Connecticut
Source: Sci Rep. 2021 Oct 12;11:20271. doi: 10.1038/s41598-021-99590-5 (PMC8511264; doi:10.1038/s41598-021-99590-5)
Supplement: Supplementary file 1 — Supplementary Information. [file 41598_2021_99590_MOESM1_ESM.pdf]

Supplement to “One year of modeling and forecasting COVID-19 transmission to support policymakers in Connecticut”

Olga Morozova[1], Zehang Richard Li[2], Forrest W. Crawford[3,4,5,6]

1. Program in Public Health and Department of Family, Population and Preventive Medicine, Stony Brook University (SUNY), NY, USA.
2. Department of Statisitcs, University of California, Santa Cruz, Santa Cruz, CA, USA.
3. Department of Biostatistics, Yale School of Public Health, New Haven, CT, USA.
4. Department of Statistics & Data Science, Yale University, New Haven, CT, USA.
5. Department of Ecology & Evolutionary Biology, Yale University, New Haven, CT, USA.
6. Yale School of Management, New Haven, CT, USA.

Estimating hospitalizations coming from non-congregate settings

Our transmission model aims to capture disease spread in the community and excludes residents of congregate settings, whose contact patterns violate homogeneous mixing assumption. Available hospitalizations data do not disaggregate by the patient’s place of residence at the time of diagnosis or hospitalization. We have therefore estimated the time-varying number of hospital admissions and hospitalizations census that came from congregate settings and excluded them from the observed time series. We received data on daily COVID-19 death counts in hospitals disaggregated by the type of residence (congregate vs. non-congregate) at the time of diagnosis or hospitalization from Connecticut Department of Public Health (CT DPH). Posterior distribution of model parameters was calibrated to the estimated time series of cumulative hospitalizations and hospitalizations census coming from non-congregate settings and to the observed time series of hospital deaths in this population.

The time-varying proportion of hospitalization census and cumulative hospitalizations coming from congregate settings was estimated as follows:

1. We first estimated the cumulative number of hospitalizations coming from congregate settings as the total number of hospital deaths among residents of congregate settings divided by the hospital case fatality ratio (HFR) among this population, and adjusting for an average hospital length of stay. The estimated HFR among residents of congregate settings in Connecticut is 0.38 and was obtained from the CT DPH based on a survey of a sample of 50 nursing homes, representing about a quarter of all nursing homes in Connecticut [1].
2. Next, we computed the cumulative proportion of hospitalizations coming from congregate settings as an estimate of the number of cumulative hospitalizations coming from congregate settings divided by the total cumulative number of hospitalizations as of the same date.
3. The proportion of hospital admissions coming from congregate settings varied over time. To approximate temporal dynamics of this proportion, we assumed that it follows the same pattern as the time-varying

proportion of deaths among hospitalized residents of congregate settings among all hospital deaths:

$$p_{\text{hospital congregate deaths}}(t) = \frac{d_{\text{hospital congregate deaths}}(t)}{d_{\text{hospital deaths}}(t)},$$

where $d(t)$ denotes smoothed daily death counts calculated as a first order difference of spline-smoothed cumulative counts of respective time series. We then re-scaled this time-varying proportion relative to the cumulative proportion of deaths among hospitalized residents of congregate settings among all hospital deaths and lagged it back by the average hospital length of stay. The resulting time-varying function was then multiplied by the cumulative proportion of hospitalizations coming from congregate settings estimated in step 2 to obtain a time-varying estimate of the proportion of hospital admissions coming from congregate settings at time t.

This time-varying proportion was then applied to the CHA-reported daily hospital census and daily hospital admissions to estimate the number of current hospitalizations, daily hospital admissions, and cumulative hospitalizations coming from congregate settings, and respective time series coming from non-congregate settings follow directly.

Data smoothing for time-varying parameters

All time-varying parameters described in detail in the Methods section rely on smoothed functions of observed data. Figure S1 shows five functions of observed data series overlaid by a smooth function (red line), which is used as a component of time-varying model parameters.

Model calibration and Bayesian posterior inference

We calibrate the posterior distribution of model parameters to the estimated statewide hospitalizations and hospital deaths coming from non-congregate settings using a Bayesian approach with a Gaussian likelihood. Model-based estimates of observed quantities are adjusted for reporting lags. The distributions of statewide hospitalizations census, cumulative hospitalizations, and hospital deaths are given by:

$$h(t) \sim \mathcal{N}\left(H(t, L_H, \boldsymbol{\theta}), \sigma_h^2\right), \tag{1}$$

$$u(t) \sim \mathcal{N}\left(U(t, L_H, \boldsymbol{\theta}), \sigma_u^2\right), \tag{2}$$

$$d(t) \sim \mathcal{N}\left(D(t, L_D, \boldsymbol{\theta}), \sigma_d^2\right), \tag{3}$$

where $H(t, L_H, \boldsymbol{\theta})$, $U(t, L_H, \boldsymbol{\theta})$, and $D(t, L_D, \boldsymbol{\theta})$ are model-projected statewide hospitalizations census (lagged by L_H), cumulative hospitalizations (lagged by L_H), and cumulative deaths (lagged by L_D) at time t under parameter values $\boldsymbol{\theta}$. Statewide model projections for a given compartment at time t are calculated as a sum of respective compartment sizes across all Connecticut counties at time t, i.e. $H(t, L_H, \boldsymbol{\theta}) = \sum_i H_i(t, L_H, \boldsymbol{\theta})$, where i indexes eight Connecticut counties. Prior distributions imposed on calibrated model parameters ensure non-negative values of model compartments. We put uniform priors on L_H and L_D over a range of plausible integer values. Reporting lags are correlated with other unknown parameters, including latency period, time between infection and hospitalization, time between infection and death, and length of hospital stay, therefore L_H and L_D should not be strictly interpreted as reporting lags. We put the same independent Inverse-Gamma(a, b) prior on all three hyperparameters σ_h^2, σ_u^2, and σ_d^2 with $a = 0.5$ and $b = 5 \times 10^6$ at the end of the modeling period. The prior was gradually relaxed as observed time series increased. For the purposes of future forecasting, as

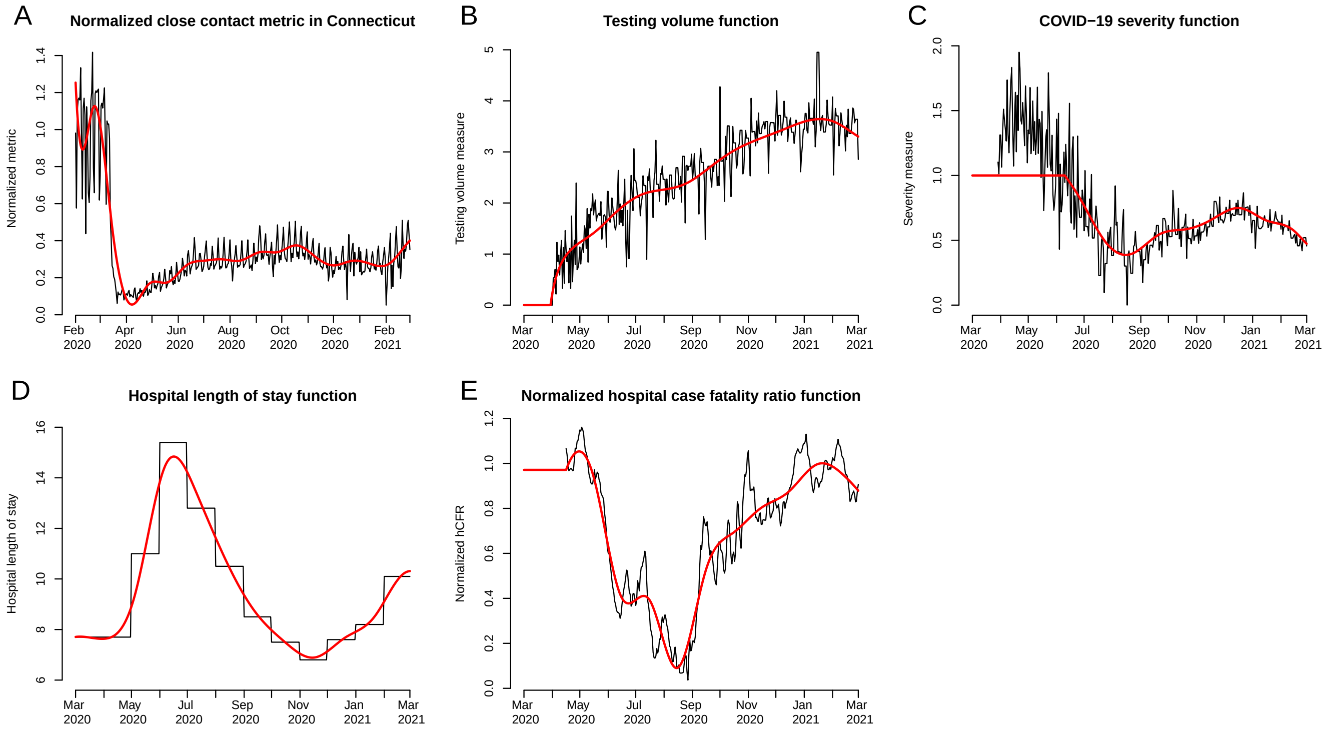

Figure S1: Smooth functions of observed data used as components of time-varying model parameters. **(A)** normalized close contact metric, function $M_{\text{contact}}(t)$; **(B)** testing volume function $M_{\text{testing}}(t)$; **(C)** COVID-19 severity function, normalized proportion of cases 60+ years old among all cases detected at time t, function $M_{\text{severity}}(t)$; **(D)** average hospital length of stay among COVID-19 patients, reciprocal of rate of hospital discharge $\gamma_H(t)$; **(E)** normalized hospital case fatality ratio, function $M_{\text{HFR}}(t)$. Black lines show input data trajectories and red lines show smooth functions of observed data.

opposed to estimation of epidemiologic features from the past model dynamics, we imposed a tighter prior on these hyperparameters to achieve reasonable posterior predictive intervals beyond the observation period.

We construct the posterior distribution over unknown parameters $(\boldsymbol{\theta}, \boldsymbol{\sigma})$ as:

$$
\begin{aligned}
p(\boldsymbol{\theta}, \boldsymbol{\sigma} | h(t), u(t), d(t)) \propto p(\boldsymbol{\theta}) p(\boldsymbol{\sigma}) & \prod_{t \in t_H} \left[p\big(h(t) | H(t, L_H, \boldsymbol{\theta}), \sigma_h\big) \right]^{w_h z(t)} \\
& \prod_{t \in t_U} \left[p\big(u(t) | U(t, L_H, \boldsymbol{\theta}), \sigma_u\big) \right]^{w_u z(t)} \\
& \prod_{t \in t_D} \left[p\big(d(t) | D(t, L_D, \boldsymbol{\theta}), \sigma_d\big) \right]^{w_d z(t)},
\end{aligned} \tag{4}
$$

where $\boldsymbol{\theta} = (\beta_0, q_A, \alpha_{I_S}, m_{H,0}, E_0, L_H, L_D, \tau, \boldsymbol{\epsilon})$ and $\boldsymbol{\sigma} = (\sigma_h, \sigma_u, \sigma_d, \sigma_\epsilon)$.

We assume the date of epidemic onset to be February 16th, 2020 - 21 days before the first case was officially confirmed in Connecticut on March 8th, 2020, and initialize the model with E_0 exposed individuals at the time of epidemic onset, setting the size of all downstream compartments to be zero. County-level distribution of E_0 is fixed and was estimated based on the county population size and dates of first registered case and death in each county.

Each likelihood term is weighted by the time-dependent weight $z(t)$ times the weight assigned to a respective time series. We let the weight function $z(t)$ take the following form,

$$
z(t) = \frac{1}{1 + \exp(-k_z t)}, \tag{5}
$$

where $z(t)$ is the weight assigned to an observation at time t, $t \in \{t_0, ..., t_{\text{max}}\}$, and the correspondence between $\{t_0, ..., t_{\text{max}}\}$ and calendar time is set such that $t = 0$ corresponds to 90 days prior to the most recent observation. Parameter k_z controls the smoothness of logistic function. We set $k_z = 0.01$, resulting in a range of weights between $0.06 - 0.7$ for the duration of observation period.

We set $w_h = 0.89$, $w_u = 0.01$, and $w_d = 0.1$. We place a large weight on the hospitalizations census, since this time series is most sensitive to changes in epidemic dynamics, and a small weight on cumulative hospitalizations, since it measures a feature that is related to hospitalizations census. The range of observation times differ for different time series. For hospitalizations census and deaths, observation times start with the first non-zero observation. For cumulative hospitalizations, observation times start on May 29, 2020 when these data started being reported routinely.

Posterior sampling

Sampling from the joint posterior distribution of $(\boldsymbol{\theta}, \boldsymbol{\sigma})$ given in (4) is performed using Markov Chain Monte Carlo (MCMC). We employ a hybrid algorithm that combines elliptical slice sampling (ESS) [2], Gibbs sampling, and Metropolis-Hastings sampling with random walk proposals. We first provide an overview of the steps in drawing samples from the full posterior distribution and then describe each steps in more details. Let $\boldsymbol{\theta} = (\boldsymbol{\theta}_{\text{MH}}, \boldsymbol{\theta}_{\text{ESS}})$, where $\boldsymbol{\theta}_{\text{MH}} = (\beta_0, q_A, \alpha_{I_S}, m_{H,0}, E_0, L_H, L_D)$ and $\boldsymbol{\theta}_{\text{ESS}} = (\tau, \boldsymbol{\epsilon})$. The sampler proceeds with the following steps:

1. Update $\boldsymbol{\theta}_{\text{ESS}} | \boldsymbol{\theta}_{\text{MH}}, \boldsymbol{\sigma}$ using ESS.
2. Update $\boldsymbol{\theta}_{\text{MH}} | \boldsymbol{\theta}_{\text{ESS}}, \boldsymbol{\sigma}$ with a Metropolis-Hastings step.
3. Update hyperparameters $\boldsymbol{\sigma} | \boldsymbol{\theta}$ with a Gibbs sampler step.

Update $\boldsymbol{\theta}_{\text{ESS}}|\boldsymbol{\theta}_{\text{MH}}, \boldsymbol{\sigma}$: We use a rejection-free sampler (ESS) to sample a vector of random effects $\boldsymbol{\epsilon}$ and a testing effect τ. The ESS operates by drawing samples from the ellipse defined by a Gaussian prior, and then accept or reject the samples by evaluating the likelihood component. Within the slice sampling step, the sampler moves along the generated ellipse and always accepts a new set of parameters.

Update $\boldsymbol{\theta}_{\text{MH}}|\boldsymbol{\theta}_{\text{ESS}}, \boldsymbol{\sigma}$: We implement a Metropolis-Hastings algorithm with random walk proposals for $\boldsymbol{\theta}_{\text{MH}}$. Proposals for L_H and L_D are made on a subset of integers bounded by a prior distribution on lags. All other elements of $\boldsymbol{\theta}_{\text{MH}}$ are continuous.

Update hyperparameters $\boldsymbol{\sigma}|\boldsymbol{\theta}$: The hyperparameters σ_h^2, σ_u^2, σ_d^2, σ_ϵ^2 are updated with Gibbs sampler steps:

$$\frac{1}{\sigma_h^2}|h(t), H(t) \sim \text{Gamma}\left(a + \frac{w_h \sum_{t \in t_H} z(t)}{2}, b + \frac{w_h \sum_{t \in t_H} z(t)(h(t) - H(t))^2}{2}\right)$$
$$\frac{1}{\sigma_u^2}|u(t), U(t) \sim \text{Gamma}\left(a + \frac{w_u \sum_{t \in t_U} z(t)}{2}, b + \frac{w_u \sum_{t \in t_U} z(t)(u(t) - U(t))^2}{2}\right)$$
$$\frac{1}{\sigma_d^2}|d(t), D(t) \sim \text{Gamma}\left(a + \frac{w_d \sum_{t \in t_D} z(t)}{2}, b + \frac{w_d \sum_{t \in t_D} z(t)(d(t) - D(t))^2}{2}\right)$$
$$\frac{1}{\sigma_\epsilon^2}|\epsilon_1, ..., \epsilon_K \sim \text{Gamma}\left(a_\epsilon + \frac{K}{2}, b_\epsilon + \frac{\sum_{i=1}^K (\epsilon_i - \epsilon_{i-1})^2}{2}\right)$$

We ran 10 chains of the sampler for 22,000 iterations each, discarded the first 2,000 draws from each chain, and thinned each chain by a factor of 20. The final posterior sample combines the resulting thinned chains. Based on the visual inspection of individual parameter trace plots, we found that 10,000 iterations is sufficient for the chain to converge in practice. Figure S2 illustrates model calibration results and convergence. The top row shows each of 10 thinned MCMC sampler chains overlaid. The middle row shows posterior histograms, marginal means and 95% posterior credible intervals of calibrated model parameters. The bottom row shows calibrated random effects function $B(t)$. To generate uncertainty intervals of model projections, we sample from the joint posterior over estimated parameters, and find pointwise 90% or 95% posterior predictive intervals for each time point.

Prior and posterior distributions of model parameters

Let $\boldsymbol{\theta}_{\text{MH,CONT}}$ denote the subset of continuous transmission model parameters whose joint distribution is calibrated to observed data and updated with Metropolis-Hastings step: $\boldsymbol{\theta}_{\text{MH, CONT}} = (\beta_0, q_A, \alpha_{I_S}, m_{H,0}, E_0)$. For each individual parameter $\theta \in \boldsymbol{\theta}_{\text{MH,CONT}}$, we specify a fixed support $[\theta_{\text{min}}, \theta_{\text{max}}]$, and put independent beta priors on the transformed parameter, i.e.,

$$\frac{\theta - \theta_{\text{min}}}{\theta_{\text{max}} - \theta_{\text{min}}} \sim \text{Beta}(a_\theta, b_\theta), \tag{6}$$

where the shape parameters a_θ and b_θ are set to let θ have mean μ_θ and standard deviation σ_θ. Table S1 provides the summary of $(\mu_\theta, \sigma_\theta, \theta_{\text{min}}, \theta_{\text{max}})$ for all parameters in $\boldsymbol{\theta}_{\text{MH,CONT}}$ along with data sources. For parameters whose values were fixed, only the mean is given. Parameter τ is updated in the ESS step, therefore in calibration, we assume a Gaussian prior distribution on $\log(\tau)$, whose mean and SD are shown in Table S1.

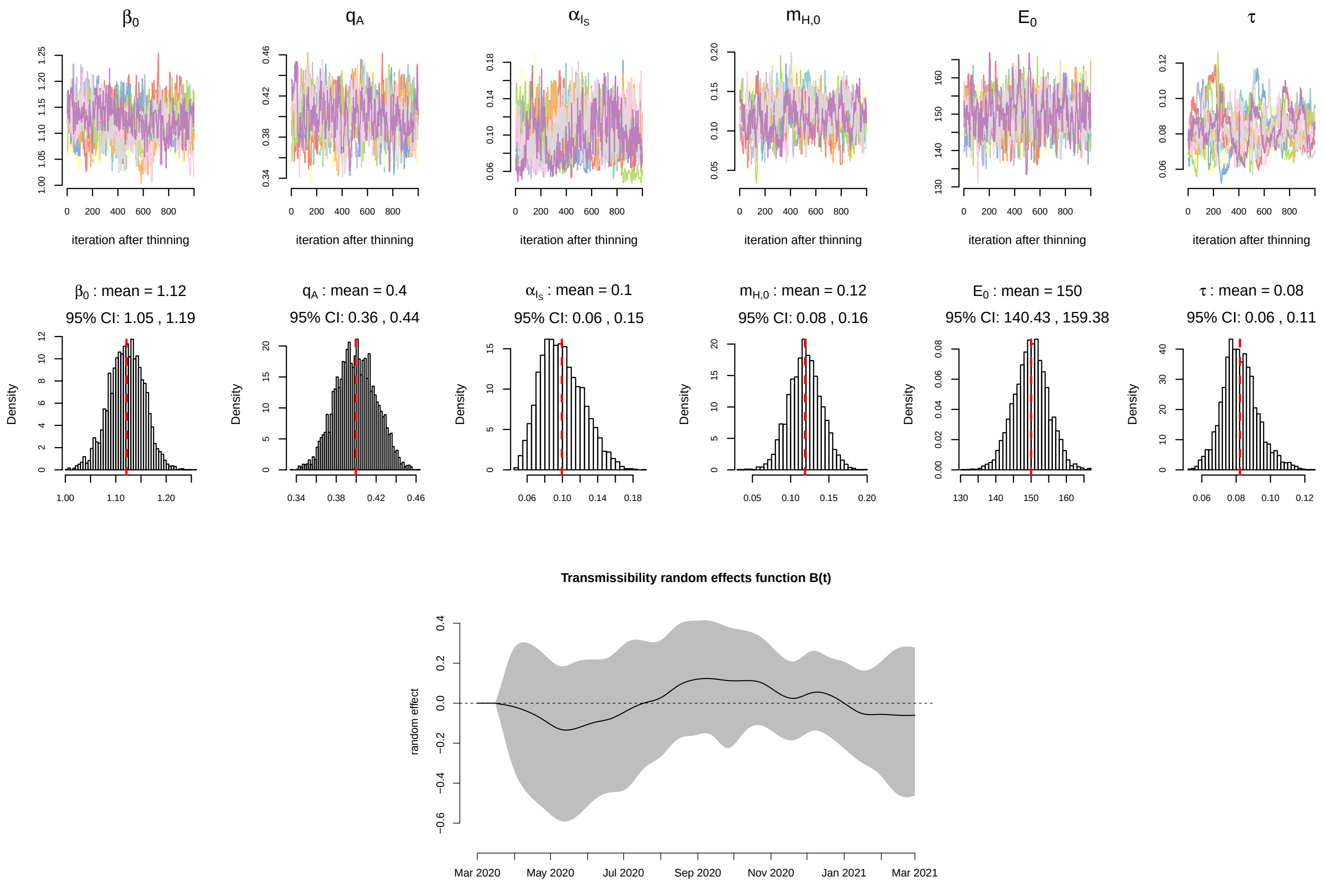

Figure S2: Model calibration results: trace plots of MCMC sampler chains, marginal posterior histograms of calibrated parameters, and random effects function $B(t)$. MCMC trace plots show 10 thinned chains plotted using different colors. Plot titles of posterior histograms include marginal posterior means and 95% credible intervals (CI). Dashed red lines correspond to posterior means of model parameters. In the transmissibility random effects function plot, black line shows the mean value of $B(t)$ at each time point, and the shaded region shows 95% CI.

Table S1: Prior distributions of model parameters.

Parameter	Mean	SD	Lower	Upper	Source
q_A	0.4	0.02	0.3	0.5	[3–8]
$q_{I_S,0}/(1-q_A)$	0.07	-	-	-	7% of symptomatic infections are severe [9–11]
β_0	1	0.5	0.01	2.00	A diffuse prior assumed
δ	1/4	-	-	-	[5, 10, 12–22]
$\alpha_{A,0}$	1/7	-	-	-	[14, 23–28]
k_A	0.5	-	-	-	[4, 13, 25, 29, 30]
$\alpha_{I_M,0}$	1/4	-	-	-	[9, 10, 12]; of 4 days, 2 are assumed to be presymptomatic [13–15]
γ_{R_M}	1/7	-	-	-	[23, 27, 28, 31]
α_{I_S}	1/9	0.03	0.02	0.2	[13–15, 32, 33]
$m_{H,0}$	0.13	0.05	0.01	0.25	Estimated from Connecticut COVID-19 hospitalization data [34]
$m_{\bar{H}}/m_H$	1.5	0.25	1	2	Assumed
$\log(\tau)$	-2.5	0.12	-	-	Assumed, Gaussian prior distribution
k_n	0.015	-	-	-	Assumed
E_0	150	5	100	200	Assumed
L_H	-	-	5	14	Assumed, uniform prior between lower and upper values
L_D	-	-	5	14	Assumed, uniform prior between lower and upper values

Asymptomatic infections play an important role in transmission of SARS-CoV-2 [35–37], but estimates of the proportion of infections that do not exhibit symptoms vary substantially [3, 4, 38]. The true proportion of asymptomatic infections is important for projections and policy planning due to its relationship to evolving herd immunity. Reported estimates of asymptomatic proportion range between 6 – 96%, and the authors of a recent review recommend a range between 40 – 45% [3]. Another review reported an overall asymptomatic proportion estimate of 20%, and 31% among the studies that included follow-up [4]. Large population-based studies conducted in Spain and in the U.K. provide estimates of asymptomatic proportion between 22-36% [39, 40]. We calculated age-adjusted weighted average of several estimates available from the literature: Nishiura et al. [6] estimated 30.8% among Japanese citizens evacuated from Wuhan, China. We applied this estimate to age group 20-64 years old. Mizumoto et al. [7] estimated 17.9% among infections on the Diamond Princess cruise ship. We applied this estimate to age group 65 plus years old, which is the age group, in which most infections occurred. We assumed 65% for age group 0-19 years old, consistent with findings reported by Russell et al. [8], where 4 out of 6 infections in this age group were asymptomatic among passengers of the Diamond Princess. The average was weighted by the age distribution of Connecticut population, resulting in the estimate of $q_A = 0.37$, which is consistent with systematic reviews and population-based studies. At the time of this writing, the best estimate of asymptomatic proportion recommended by the CDC was 40% [5]. We center the prior distribution of q_A at 0.4 and allow it to vary between 0.3 and 0.5. The proportion of severe cases early in the epidemic ($q_{I_S,0}$) is assumed to be 7% of symptomatic infections in line with [9–11]. Combined with the estimates of asymptomatic proportion, this translates into an estimate of severe proportion of 4.2% (3.5-4.9%) of all infections.

Duration of latency ($1/\delta$) was fixed, since consistent estimates of this parameter are available from the literature,

and because it is highly correlated with several calibrated parameters, including reporting lags and parameters that determine duration of infectiousness and time between infection and recovery or death. We assume an average of 4 days of latency [10, 12, 13] and 2 days of presymptomatic infectiousness [13–15] resulting in an average incubation period of 6 days consistent with [5, 16–22].

Parameters (α_A, k_A, α_{I_M}, α_{I_S}) collectively determine the force of infection at any given time. Force of infection, transmission parameter β and initial number of exposed individuals E_0 together determine the early growth of the epidemic. Without additional data, all of these parameters cannot be simultaneously identified. We therefore fixed the values for a subset of these parameters based on available estimates and assumed a diffuse prior on β, which absorbs additional variation of parameters that determine the force of infection.

Duration of infectiousness of asymptomatic individuals is unknown, but is likely shorter than that of symptomatic individuals [23–26]. While several studies estimated that viral RNA could be detected in upper respiratory tract for 2-3 weeks [41, 42], findings from [23, 27, 28] show that in mild-to-moderate symptomatic patients live virus could be isolated for a substantially shorter time period: up to 7-10 days from the day of symptom onset, suggesting the duration of infectiousness of up to 12 days. Based on these estimates and [14], we assume the duration of infectiousness of 7 days among asymptomatic individuals. Although multiple studies have shown similar viral loads among symptomatic, presymptomatic and asymptomatic cases [24, 43, 44], evidence suggests that asymptomatic individuals are less infectious that symptomatic, likely due to higher viral shedding while coughing and longer duration of infectiousness among symptomatic individuals [4, 23, 25, 45, 46]. Multiple estimates of relative infectiousness of asymptomatic individuals compared to symptomatic are available and range from close to zero to above one [4, 13, 25, 29, 30]. Generally, estimates that are based on attack rates are lower than those based on viral shedding or time until the first negative PCR test. In our analysis, we set relative infectiousness of asymptomatic cases to be 0.5 [4, 13, 29].

Although the duration of infectiousness of mild-to-moderately symptomatic cases may be up to 12 days [23, 27, 28], we assume that the majority of symptomatic individuals self-isolate shortly after developing symptoms. We set the duration of infectiousness of symptomatic cases to be 4 days [9, 10, 12], 2 of which are assumed to represent presymptomatic infectiousness [13–15]. We further assume that the duration of self-isolation until recovery is 7 days [23, 27, 28].

We calibrate the rate of hospitalization among severe cases (α_{I_S}), assuming a mean of 9 days between the onset of infectiousness and hospitalization: 2 days of presymptomatic infectiousness plus 7 days between the onset of symptoms and hospitalization [32, 33].

Definitions and estimation of epidemiologic parameters

We compute model-based estimates of the following epidemiologic parameters that describe SARS-CoV-2 transmission and COVID-19 disease progression:

Basic (R_0) and effective (R_{eff}) reproduction number

Basic reproduction number is defined as the expected number of people that a single infected individual will infect before recovery or death in a fully susceptible population. It is usually estimated at the beginning of an epidemic before any interventions intended to slow down transmission are implemented. Effective reproduction number has the same meaning, but adjusts for depletion of susceptible individuals and, potentially, effects of transmission-reducing interventions. It shows the expected number of people that a single infected individual

will infect at time t as the epidemic progresses. We compute a model-based estimate of R_{eff} as follows:

$$R_{\text{eff}}(t) = \beta(t) \left(\frac{q_A k_A}{\alpha_A(t)} + \frac{q_{I_S}(t)}{\alpha_{I_S}} + \frac{1 - q_A - q_{I_S}(t)}{\alpha_{I_M}(t)} \right) \frac{S(t)}{N - D(t)},$$

where expressions for time-varying model parameters are provided in the Methods section of the main text, $S(t)$ and $D(t)$ are statewide numbers of susceptible and cumulative deceased individuals at time t, and N is the size of Connecticut population residing in non-congregate settings. We estimate R_0 using the formula for R_{eff} at time zero (March 1, 2020).

Instantaneous and cumulative case-detection ratio (CDR)

CDR is defined as a proportion of all infections, including asymptomatic, that are detected and become known to the public health surveillance system. We estimate instantaneous CDR at time t as:

$$\text{CDR}(t) = \frac{\text{reported cases } (t)}{\delta E(t - L_I)},$$

where reported cases at time t represent a 7-day moving average of officially diagnosed and reported COVID-19 cases in Connecticut residing in non-congregate settings, and $\delta E(t - L_I)$ is a model-projected statewide number of new infections in Connecticut at time $t - L_I$, where L_I is an infection detection and reporting lag set to 14 days. Cumulative CDR is estimated as a sum of all detected COVID-19 cases residing in non-congregate settings since the epidemic onset through time T (March 1, 2021) divided by the model-projected cumulative number of infections since the epidemic onset through time $T - L_I$.

Infection hospitalization ratio (IHR)

IHR is defined as a proportion of all infections, including asymptomatic, which get admitted to a hospital. We compute an estimate of cumulative IHR as a ratio of reported cumulative hospitalizations to a model-based projection of a sum of hospitalized, recovered, and deceased individuals, adjusting for reporting lag L_H.

Infection fatality ratio (IFR)

IFR is defined as a proportion of all infections, including asymptomatic, which result in death. We compute an estimate of cumulative IFR as a ratio of reported cumulative deaths to a model-based projection of a sum of recovered and deceased individuals, adjusting for reporting lag L_D.

Hospital case fatality ratio (HFR)

HFR is defined as a proportion of patients admitted to a hospital who die. We compute instantaneous HFR at time t as a ratio of hospital deaths at time t to hospital admissions at time $t -$ HLOS(t), where HLOS(t) is an average hospital length of stay at time t.

References

[1] State of Connecticut Office of Policy and Management. Connecticut Open Data: COVID-19 Data Resources. https://data.ct.gov/stories/s/COVID-19-data/wa3g-tfvc/. Accessed: 2021-03-29.

[2] Iain Murray, Ryan Adams, and David MacKay. Elliptical slice sampling. In *Proceedings of the thirteenth international conference on artificial intelligence and statistics*, pages 541–548, 2010.

[3] Daniel P Oran and Eric J Topol. Prevalence of asymptomatic SARS-CoV-2 infection: a narrative review. *Annals of Internal Medicine*, 173(5):362–367, 2020.

[4] Diana Buitrago-Garcia, Dianne Egli-Gany, Michel J Counotte, Stefanie Hossmann, Hira Imeri, Aziz Mert Ipekci, Georgia Salanti, and Nicola Low. Occurrence and transmission potential of asymptomatic and presymptomatic SARS-CoV-2 infections: A living systematic review and meta-analysis. *PLoS medicine*, 17(9):e1003346, 2020.

[5] Centers for Disease Control and Prevention. COVID-19 Pandemic Planning Scenarios. https://www.cdc.gov/coronavirus/2019-ncov/hcp/planning-scenarios.html, 2021. Accessed: 2021-01-18.

[6] Hiroshi Nishiura, Tetsuro Kobayashi, Takeshi Miyama, Ayako Suzuki, Sung-Mok Jung, Katsuma Hayashi, Ryo Kinoshita, Yichi Yang, Baoyin Yuan, Andrei R Akhmetzhanov, and Natalie M Linton. Estimation of the asymptomatic ratio of novel coronavirus infections (COVID-19). *International Journal of Infectious Diseases*, 94:154 – 155, 2020.

[7] Kenji Mizumoto, Katsushi Kagaya, Alexander Zarebski, and Gerardo Chowell. Estimating the asymptomatic proportion of coronavirus disease 2019 (COVID-19) cases on board the Diamond Princess cruise ship, Yokohama, Japan, 2020. *Eurosurveillance*, 25(10):2000180, 2020.

[8] Timothy W Russell, Joel Hellewell, Christopher I Jarvis, Kevin Van Zandvoort, Sam Abbott, Ruwan Ratnayake, Stefan Flasche, Rosalind M Eggo, W John Edmunds, and Adam J Kucharski. Estimating the infection and case fatality ratio for coronavirus disease (COVID-19) using age-adjusted data from the outbreak on the Diamond Princess cruise ship, February 2020. *Eurosurveillance*, 25(12):2000256, 2020.

[9] Stephen M Kissler, Christine Tedijanto, Edward Goldstein, Yonatan H Grad, and Marc Lipsitch. Projecting the transmission dynamics of SARS-CoV-2 through the postpandemic period. *Science*, 368(6493):860–868, 2020.

[10] Henrik Salje, Cécile Tran Kiem, Noémie Lefrancq, Noémie Courtejoie, Paolo Bosetti, Juliette Paireau, Alessio Andronico, Nathanaël Hozé, Jehanne Richet, Claire-Lise Dubost, Yann Le Strat, Justin Lessler, Daniel Levy Bruhl, Arnaud Fontanet, Lulla Opatowski, Pierre-Yves Boelle, and Simon Cauchemez. Estimating the burden of SARS-CoV-2 in France. *Science*, 369(6500):208–211, 2020.

[11] Robert Verity, Lucy C Okell, Ilaria Dorigatti, Peter Winskill, Charles Whittaker, Natsuko Imai, Gina Cuomo-Dannenburg, Hayley Thompson, Patrick GT Walker, Han Fu, Amy Dighe, Jamie T Griffin, Marc Baguelin, Sangeeta Bhatia, Anne Boonyasiri, Adhiratha andd Cori, Zulma Cucunubá, Rich FitzJohn, Katy Gaythorpe, Will Green, Arran Hamlet, Wes Hinsley, Daniel Laydon, Gemma Nedjati-Gilani, Steven Riley, Sabine van Elsland, Erik Volz, Haowei Wang, Yuanrong Wang, Xiaoyue Xi, Christl A Donnelly, Azra C Ghani, and Neil M Ferguson. Estimates of the severity of coronavirus disease 2019: a model-based analysis. *The Lancet Infectious Diseases*, 20(6):669–677, 2020. doi: https://doi.org/10.1016/S1473-3099(20)30243-7.

[12] Ruiyun Li, Sen Pei, Bin Chen, Yimeng Song, Tao Zhang, Wan Yang, and Jeffrey Shaman. Substantial undocumented infection facilitates the rapid dissemination of novel coronavirus (SARS-CoV2). *Science*, 368(6490):489–493, 2020. doi: 10.1126/science.abb3221.

[13] Alberto Aleta, David Martin-Corral, Ana Pastore y Piontti, Marco Ajelli, Maria Litvinova, Matteo Chinazzi, Natalie E Dean, M Elizabeth Halloran, Ira M Longini Jr, Stefano Merler, Alex Pentland, Alessandro Vespignani, Esteban Moro, and Yamir Moreno. Modelling the impact of testing, contact tracing and household quarantine on second waves of COVID-19. *Nature Human Behaviour*, 4(9):964–971, 2020. doi: https://doi.org/10.1038/s41562-020-0931-9.

[14] Andrew William Byrne, David McEvoy, Aine B Collins, Kevin Hunt, Miriam Casey, Ann Barber, Francis

Butler, John Griffin, Elizabeth A Lane, Conor McAloon, Kirsty O'Brien, Patrick Wall, Kieran A Walsh, and Simon J More. Inferred duration of infectious period of SARS-CoV-2: rapid scoping review and analysis of available evidence for asymptomatic and symptomatic COVID-19 cases. *BMJ Open*, 10(8), 2020. ISSN 2044-6055. doi: 10.1136/bmjopen-2020-039856. URL `https://bmjopen.bmj.com/content/10/8/e039856`.

[15] Wycliffe E Wei, Zongbin Li, Calvin J Chiew, Sarah E Yong, Matthias P Toh, and Vernon J Lee. Presymptomatic transmission of SARS-CoV-2 – Singapore, January 23–March 16, 2020. *Morbidity and Mortality Weekly Report*, 69(14):411, 2020.

[16] Matthew Biggerstaff, Benjamin J Cowling, Zulma M Cucunubá, Linh Dinh, Neil M Ferguson, Huizhi Gao, Verity Hill, Natsuko Imai, Michael A Johansson, Sarah Kada, Oliver Morgan, Ana Pastore y Piontti, Jonathan A. Polonsky, Pragati Venkata Prasad, Talia M. Quandelacy, Andrew Rambaut, Jordan W. Tappero, Katelijn A. Vandemaele, Alessandro Vespignani, K. Lane Warmbrod, Jessica Y. Wong, and the WHO COVID-19 Modelling Parameters Group. Early insights from statistical and mathematical modeling of key epidemiologic parameters of COVID-19. *Emerging Infectious Diseases*, 26(11), 2020. doi: https://doi.org/10.3201/eid2611.201074.

[17] Conor McAloon, Áine Collins, Kevin Hunt, Ann Barber, Andrew W Byrne, Francis Butler, Miriam Casey, John Griffin, Elizabeth Lane, David McEvoy, Patrick Wall, Martin Green, Luke O'Grady, and Simon J More. Incubation period of COVID-19: a rapid systematic review and meta-analysis of observational research. *BMJ Open*, 10(8):e039652, 2020. doi: 10.1136/bmjopen-2020-039652.

[18] Stephen A Lauer, Kyra H Grantz, Qifang Bi, Forrest K Jones, Qulu Zheng, Hannah R Meredith, Andrew S Azman, Nicholas G Reich, and Justin Lessler. The incubation period of coronavirus disease 2019 (COVID-19) from publicly reported confirmed cases: estimation and application. *Annals of Internal Medicine*, 172 (9):577–582, 2020.

[19] Jantien A Backer, Don Klinkenberg, and Jacco Wallinga. Incubation period of 2019 novel coronavirus (2019-nCoV) infections among travellers from Wuhan, China, 20–28 January 2020. *Eurosurveillance*, 25 (5):2000062, 2020.

[20] Qun Li, Xuhua Guan, Peng Wu, Xiaoye Wang, Lei Zhou, Yeqing Tong, Ruiqi Ren, Kathy S.M. Leung, Eric H.Y. Lau, Jessica Y. Wong, Xuesen Xing, Nijuan Xiang, Yang Wu, Chao Li, Qi Chen, Dan Li, Tian Liu, Jing Zhao, Man Liu, Wenxiao Tu, Chuding Chen, Lianmei Jin, Rui Yang, Qi Wang, Suhua Zhou, Rui Wang, Hui Liu, Yinbo Luo, Yuan Liu, Ge Shao, Huan Li, Zhongfa Tao, Yang Yang, Zhiqiang Deng, Boxi Liu, Zhitao Ma, Yanping Zhang, Guoqing Shi, Tommy T.Y. Lam, Joseph T. Wu, George F. Gao, Benjamin J. Cowling, Bo Yang, Gabriel M. Leung, and Zijian Feng. Early transmission dynamics in Wuhan, China, of novel coronavirus–infected pneumonia. *New England Journal of Medicine*, 382(13):1199–1207, 2020. doi: 10.1056/NEJMoa2001316.

[21] Natalie M Linton, Tetsuro Kobayashi, Yichi Yang, Katsuma Hayashi, Andrei R Akhmetzhanov, Sung-mok Jung, Baoyin Yuan, Ryo Kinoshita, and Hiroshi Nishiura. Incubation period and other epidemiological characteristics of 2019 novel coronavirus infections with right truncation: a statistical analysis of publicly available case data. *Journal of Clinical Medicine*, 9(2):538, 2020.

[22] Jing Qin, Chong You, Qiushi Lin, Taojun Hu, Shicheng Yu, and Xiao-Hua Zhou. Estimation of incubation period distribution of COVID-19 using disease onset forward time: A novel cross-sectional and forward follow-up study. *Science Advances*, 6(33), 2020. doi: 10.1126/sciadv.abc1202. URL `https://advances.sciencemag.org/content/6/33/eabc1202`.

[23] Muge Cevik, Matthew Tate, Ollie Lloyd, Alberto Enrico Maraolo, Jenna Schafers, and Antonia Ho. SARS-CoV-2, SARS-CoV, and MERS-CoV viral load dynamics, duration of viral shedding, and infectiousness: a systematic review and meta-analysis. *The Lancet Microbe*, 2(1):e13–e22, 2021. doi: https://doi.org/10.1016/S2666-5247(20)30172-5.

[24] Kieran A Walsh, Karen Jordan, Barbara Clyne, Daniela Rohde, Linda Drummond, Paula Byrne, Susan Ahern, Paul G Carty, Kirsty K O'Brien, Eamon O'Murchu, Michelle O'Neill, Susan M Smith, Mairin Ryan, and Patricia Harrington. SARS-CoV-2 detection, viral load and infectivity over the course of an infection. *Journal of Infection*, 81(3):357–371, 2020. doi: https://doi.org/10.1016/j.jinf.2020.06.067.

[25] Xueting Qiu, Ali Ihsan Nergiz, Alberto Enrico Maraolo, Isaac I. Bogoch, Nicola Low, and Muge Cevik. The role of asymptomatic and pre-symptomatic infection in SARS-CoV-2 transmission — a living systematic review. *Clinical Microbiology and Infection*, 27(4):511–519, 2021. doi: https://doi.org/10.1016/j.cmi.2021.01.011.

[26] Rongrong Yang, Xien Gui, and Yong Xiong. Comparison of clinical characteristics of patients with asymptomatic vs symptomatic coronavirus disease 2019 in Wuhan, China. *JAMA Network Open*, 3(5): e2010182–e2010182, 2020.

[27] Kieran A Walsh, Susan Spillane, Laura Comber, Karen Cardwell, Patricia Harrington, Jeff Connell, Conor Teljeur, Natasha Broderick, Cillian F de Gascun, Susan M Smith, Mairin Ryan, and Michelle O'Neill. The duration of infectiousness of individuals infected with SARS-CoV-2. *Journal of Infection*, 81(6):847–856, 2020. doi: https://doi.org/10.1016/j.jinf.2020.10.009.

[28] Roman Wölfel, Victor M. Corman, Wolfgang Guggemos, Michael Seilmaier, Sabine Zange, Marcel A. Müller, Daniela Niemeyer, Terry C. Jones, Patrick Vollmar, Camilla Rothe, Michael Hoelscher, Tobias Bleicker, Sebastian Brünink, Julia Schneider, Rosina Ehmann, Katrin Zwirglmaier, Christian Drosten, and Clemens Wendtner. Virological assessment of hospitalized patients with COVID-2019. *Nature*, 581(7809): 465–469, 2020.

[29] David Mc Evoy, Conor G McAloon, Aine B Collins, Kevin Hunt, Francis Butler, Andrew W Byrne, Miriam Casey, Ann Barber, John M Griffin, Elizabeth A Lane, Patrick Wall, and Simon J More. The relative infectiousness of asymptomatic SARS-CoV-2 infected persons compared with symptomatic individuals: A rapid scoping review. *medRxiv*, 2020. doi: https://doi.org/10.1101/2020.07.30.20165084. URL https://www.medrxiv.org/content/10.1101/2020.07.30.20165084v1.

[30] Daihai He, Shi Zhao, Qianying Lin, Zian Zhuang, Peihua Cao, Maggie H Wang, and Lin Yang. The relative transmissibility of asymptomatic COVID-19 infections among close contacts. *International Journal of Infectious Diseases*, 94:145–147, 2020.

[31] Centers for Disease Control and Prevention. Discontinuation of isolation for persons with COVID-19 not in healthcare settings. interim guidance. https://www.cdc.gov/coronavirus/2019-ncov/hcp/disposition-in-home-patients.html, 2020. Accessed: 2021-01-18.

[32] Shikha Garg, Lindsay Kim, Michael Whitaker, Alissa O'Halloran, Charisse Cummings, Rachel Holstein, Mila Prill, Shua J Chai, Pam D Kirley, Nisha B Alden, Breanna Kawasaki, Kimberly Yousey-Hindes, Linda Niccolai, Evan J Anderson, Kyle P Openo, Andrew Weigel, Maya L Monroe, Patricia Ryan, Justin Henderson, Sue Kim, Kathy Como-Sabetti, Ruth Lynfield, Daniel Sosin, Salina Torres, Alison Muse, Nancy M Bennett, Laurie Billing, Melissa Sutton, Nicole West, William Schaffner, H. Keipp Talbot, Aquino Clarissa, Andrea George, Alicia Budd, Lynnette Brammer, Gayle Langley, Aron J Hall, and Alicia Fry. Hospitalization rates and characteristics of patients hospitalized with laboratory-confirmed coronavirus disease 2019 – COVID-NET, 14 states, March 1–30, 2020. *MMWR. Morbidity and Mortality Weekly Report*, 69(15): 458–464, 2020. doi: 10.15585/mmwr.mm6915e3.

[33] Pablo N Perez-Guzman, Anna Daunt, Sujit Mukherjee, Peter Crook, Roberta Forlano, Mara D Kont, Alessandra Løchen, Michaela Vollmer, Paul Middleton, Rebekah Judge, , Chris Harlow, Anet Soubieres, Graham Cooke, Peter J White, Timothy B Hallett, Paul Aylin, Neil Ferguson, Katharina Hauck, Mark Thursz, and Shevanthi Nayagam. Clinical characteristics and predictors of outcomes of hospitalized patients with coronavirus disease 2019 in a multiethnic London national health service trust: a retrospective cohort study. *Clinical Infectious Diseases*, ciaa1091, 2020. doi: 10.1093/cid/ciaa1091.

[34] Connecticut Hospital Association. URL https://cthosp.org/.

[35] Nathan W Furukawa, John T Brooks, and Jeremy Sobel. Evidence supporting transmission of severe acute respiratory syndrome coronavirus 2 while presymptomatic or asymptomatic. *Emerging Infectious Diseases*, 26(7), 2020.

[36] Jon C Emery, Timothy W Russel, Yang Liu, Joel Hellewell, Carl AB Pearson, CMMID COVID-19 Working Group, Gwen M Knight, Rosalind M Eggo, Adam J Kucharski, Sebastian Funk, Stefan Flasche, and Rein MGJ Houben. The contribution of asymptomatic SARS-CoV-2 infections to transmission on the Dia-

mond Princess cruise ship. *eLife*, 9(e58699), 2020. doi: 10.7554/eLife.58699.

[37] Xi He, Eric H. Y. Lau, Peng Wu, Xilong Deng, Jian Wang, Xinxin Hao, Yiu Chung Lau, Jessica Y. Wong, Yujuan Guan, Xinghua Tan, Xiaoneng Mo, Yanqing Chen, Baolin Liao, Weilie Chen, Fengyu Hu, Qing Zhang, Mingqiu Zhong, Yanrong Wu, Lingzhai Zhao, Fuchun Zhang, Benjamin J. Cowling, Fang Li, and Gabriel M. Leung. Temporal dynamics in viral shedding and transmissibility of COVID-19. *Nature Medicine*, 26(5):672–675, 2020.

[38] Mercedes Yanes-Lane, Nicholas Winters, Federica Fregonese, Mayara Bastos, Sara Perlman-Arrow, Jonathon R Campbell, and Dick Menzies. Proportion of asymptomatic infection among COVID-19 positive persons and their transmission potential: A systematic review and meta-analysis. *PloS one*, 15(11): e0241536, 2020.

[39] Marina Pollán, Beatriz Pérez-Gómez, Roberto Pastor-Barriuso, Jesús Oteo, Miguel A Hernán, Mayte Pérez-Olmeda, Jose L Sanmartín, Aurora Fernández-García, Israel Cruz, Nerea Fernández de Larrea, et al. Prevalence of SARS-CoV-2 in Spain (ENE-COVID): a nationwide, population-based seroepidemiological study. *The Lancet*, 396(10250):535–544, 2020.

[40] Helen Ward, Christina J Atchison, Matthew Whitaker, Kylie E. C. Ainslie, Joshua Elliott, Lucy C Okell, Rozlyn Redd, Deborah Ashby, Christl A. Donnelly, Wendy Barclay, Ara Darzi, Graham Cooke, Steven Riley, and Paul Elliott. Antibody prevalence for SARS-CoV-2 following the peak of the pandemic in England: REACT2 study in 100,000 adults. *medRxiv*, 2020. doi: 10.1101/2020.08.12.20173690. URL `https://www.medrxiv.org/content/10.1101/2020.08.12.20173690v2`.

[41] Barnaby Edward Young, Sean Wei Xiang Ong, Shirin Kalimuddin, Jenny G. Low, Seow Yen Tan, Jiashen Loh, Oon-Tek Ng, Kalisvar Marimuthu, Li Wei Ang, Tze Minn Mak, Sok Kiang Lau, Danielle E. Anderson, Kian Sing Chan, Thean Yen Tan, Tong Yong Ng, Lin Cui, Zubaidah Said, Lalitha Kurupatham, Mark I-Cheng Chen, Monica Chan, Shawn Vasoo, Lin-Fa Wang, Boon Huan Tan, Raymond Tzer Pin Lin, Vernon Jian Ming Lee, Yee-Sin Leo, David Chien Lye, and for the Singapore 2019 Novel Coronavirus Outbreak Research Team. Epidemiologic features and clinical course of patients infected with SARS-CoV-2 in Singapore. *JAMA*, 323(15):1488–1494, 2020.

[42] The COVID-19 Investigation Team. Clinical and virologic characteristics of the first 12 patients with coronavirus disease 2019 (COVID-19) in the United States. *Nature Medicine*, 26(6):861–868, 2020. doi: https://doi.org/10.1038/s41591-020-0877-5.

[43] Enrico Lavezzo, Elisa Franchin, Constanze Ciavarella, Gina Cuomo-Dannenburg, Luisa Barzon, Claudia Del Vecchio, Lucia Rossi, Riccardo Manganelli, Arianna Loregian, Nicolò Navarin, Davide Abate, Manuela Sciro, Stefano Merigliano, Ettore De Canale, Maria Cristina Vanuzzo, Valeria Besutti, Francesca Saluzzo, Francesco Onelia, Monia Pacenti, Saverio G. Parisi, Giovanni Carretta, Daniele Donato, Luciano Flor, Silvia Cocchio, Giulia Masi, Alessandro Sperduti, Lorenzo Cattarino, Renato Salvador, Michele Nicoletti, Federico Caldart, Gioele Castelli, Eleonora Nieddu, Beatrice Labella, Ludovico Fava, Matteo Drigo, Katy A. M. Gaythorpe, Alessandra R. Brazzale, Stefano Toppo, Marta Trevisan, Vincenzo Baldo, Christl A. Donnelly, Neil M. Ferguson, Ilaria Dorigatti, Andrea Crisanti, and Imperial College COVID-19 Response Team. Suppression of a SARS-CoV-2 outbreak in the Italian municipality of Vo'. *Nature*, 584(7821): 425–429, 2020.

[44] Seungjae Lee, Tark Kim, Eunjung Lee, Cheolgu Lee, Hojung Kim, Heejeong Rhee, Se Yoon Park, Hyo-Ju Son, Shinae Yu, Jung Wan Park, Eun Ju Choo, Suyeon Park, Mark Loeb, and Tae Hyong Kim. Clinical course and molecular viral shedding among asymptomatic and symptomatic patients with SARS-CoV-2 infection in a community treatment center in the Republic of Korea. *JAMA Internal Medicine*, 180(11): 1447–1452, 2020.

[45] Muge Cevik, Krutika Kuppalli, Jason Kindrachuk, and Malik Peiris. Virology, transmission, and pathogenesis of SARS-CoV-2. *BMJ*, 371(m3862), 2020. doi: https://doi.org/10.1136/bmj.m3862.

[46] Liling Chaw, Wee Chian Koh, Sirajul Adli Jamaludin, Lin Naing, Mohammad Fathi Alikhan, and Justin Wong. Analysis of SARS-CoV-2 transmission in different settings, Brunei. *Emerging infectious diseases*, 26(11), 2020.
